# Autoinhibition of kinesin-1 is essential to the dendrite-specific localization of Golgi outposts

**DOI:** 10.1083/jcb.201708096

**Published:** 2018-07-02

**Authors:** Michael T. Kelliher, Yang Yue, Ashley Ng, Daichi Kamiyama, Bo Huang, Kristen J. Verhey, Jill Wildonger

**Affiliations:** 1 Integrated Program in Biochemistry, University of Wisconsin-Madison, Madison, WI; 2 Biochemistry Department, University of Wisconsin-Madison, Madison, WI; 3 Department of Cell and Developmental Biology, University of Michigan, Ann Arbor, MI; 4 Biochemistry Scholars Program, University of Wisconsin-Madison, Madison, WI; 5 Department of Pharmaceutical Chemistry, University of California, San Francisco, San Francisco, CA

## Abstract

Neuronal polarity relies on the selective localization of cargo to axons or dendrites. The molecular motor kinesin-1 moves cargo into axons but is also active in dendrites. This raises the question of how kinesin-1 activity is regulated to maintain the compartment-specific localization of cargo. Our in vivo structure–function analysis of endogenous *Drosophila melanogaster* kinesin-1 reveals a novel role for autoinhibition in enabling the dendrite-specific localization of Golgi outposts. Mutations that disrupt kinesin-1 autoinhibition result in the axonal mislocalization of Golgi outposts. Autoinhibition also regulates kinesin-1 localization. Uninhibited kinesin-1 accumulates in axons and is depleted from dendrites, correlating with the change in outpost distribution and dendrite growth defects. Genetic interaction tests show that a balance of kinesin-1 inhibition and dynein activity is necessary to localize Golgi outposts to dendrites and keep them from entering axons. Our data indicate that kinesin-1 activity is precisely regulated by autoinhibition to achieve the selective localization of dendritic cargo.

## Abstract (precis)

Neuronal polarity relies on the axon- or dendrite-specific localization of cargo by molecular motors such as kinesin-1. This study shows how autoinhibition regulates both kinesin-1 activity and localization to keep dendritic Golgi outposts from entering axons.

## Introduction

To receive and send signals, neurons rely on the polarized distribution of vesicles, macromolecules, and organelles to dendrites and axons. The majority of transport in neurons is mediated by the microtubule (MT)–based motors kinesin and dynein, which traffic cargo in both compartments. To investigate how motor function is regulated to maintain proper cargo distribution we focused on the MT plus end–directed kinesin-1. Kinesin-driven events must be tightly controlled in the cell body to ensure that both outgoing and incoming cargos reach their proper destinations.

In mammalian neurons, both axons and dendrites contain plus end–distal MTs. This raises the question of how kinesin-1 is regulated to achieve compartment-specific cargo distribution. One model proposes that kinesin-1 distinguishes between axonal and dendritic MTs (Atherton et al., 2013; Bentley and Banker, 2016; Tas et al., 2017). This model arose from experiments characterizing the distribution of truncated, constitutively active kinesin motors lacking C-terminal cargo-binding and autoregulatory domains. The cellular localization of truncated kinesins suggests that kinesin-1 differentiates between MT tracks patterned by posttranslational modifications and MT-associated proteins (Nakata and Hirokawa, 2003; Jacobson et al., 2006; Cai et al., 2009; Hammond et al., 2010; Nakata et al., 2011; Atherton et al., 2013; Farías et al., 2015; Guardia et al., 2016; Tas et al., 2017; Tortosa et al., 2017).

In contrast with mammalian neurons, sorting decisions in the cell body of invertebrate neurons are theoretically simplified as axons contain plus end–distal MTs whereas dendrites contain predominantly minus end–distal MTs (Rolls et al., 2007; Stone et al., 2008). However, for both vertebrate and invertebrate neurons, transport must be highly regulated to prevent kinesin-driven transport in dendrites from continuing unimpeded into axons upon reaching the cell body. Similarly, in the cell body, kinesin-1 bound to dendritic cargo must be inactivated to enable transport out to dendrites by dynein and to prevent axon entry. In both mammalian and invertebrate neurons, MT polarity must be coupled with additional mechanisms that regulate kinesin-1 activity and/or cargo attachment to achieve proper cargo distribution (Fu and Holzbaur, 2014).

Kinesin-1, like other kinesin family members, is regulated by autoinhibition (Verhey and Hammond, 2009). Autoinhibition inactivates kinesin-1 when it is not cargo-bound and may fine-tune motor activity when kinesin-1 is cargo-bound (Lane and Allan, 1999; Woźniak and Allan, 2006; Verhey and Hammond, 2009; Fu and Holzbaur, 2013; Fu et al., 2014; Maday et al., 2014). Although in vitro studies indicate that autoinhibition plays a key role in controlling motor function, the cellular role or roles for autoinhibition of kinesin-1 are still poorly understood. To date, motor autoinhibition has been shown to modulate axonal transport (kinesin-1), axon outgrowth (kinesin-3), and synapse positioning (kinesin-4; Moua et al., 2011; van der Vaart et al., 2013; Cheng et al., 2014; Niwa et al., 2016). It is attractive to consider the possibility that autoinhibition of kinesin-1 would regulate the compartment-specific localization of cargo as well.

We used in vivo structure–function analysis of endogenous kinesin-1 in fruit flies to uncover mechanisms that regulate kinesin-1. We sought to identify mutations that would lead to the axonal mislocalization of dendritic cargo, specifically Golgi outposts, which our data suggest are carried by kinesin-1. We initially tested the idea that kinesin-1 uses its MT-binding domain (MTBD) to “read” positional information encoded on MTs. Studies of truncated kinesin-1 have implicated three different loops in the kinesin-1 MTBD (β5-loop 8, loop 11, and loop 12) in polarized transport; however, there is no consensus on which loops are essential (Nakata and Hirokawa, 2003; Konishi and Setou, 2009; Nakata et al., 2011; Huang and Banker, 2012). Our in vivo studies of endogenous kinesin-1 show that E177 within β5-loop 8 is critical to the dendrite-specific localization of Golgi outposts. This particular glutamate, E177, is part of a short motif previously implicated in kinesin-1’s preference for axons in vertebrate neurons (Konishi and Setou, 2009), but it also mediates contact with the C-terminal tail when kinesin-1 is in an autoinhibited conformation (Kaan et al., 2011). Our combined in vivo and in vitro results indicate that mutating E177 results in the axonal mislocalization of Golgi outposts by relieving kinesin-1 autoinhibition. We show that autoinhibition is also critical for the proper localization of kinesin-1 itself. Like constitutively active truncated motor constructs, uninhibited full-length kinesin-1 is enriched in axon terminals and nearly depleted from dendrites, which correlates with the axonal mislocalization of outposts and reduced dendrite growth. Last, our genetic interaction tests suggest that outposts are restricted to dendrites by balancing the activities of kinesin-1 and dynein. Dynein transports outposts into dendrites (Ye et al., 2007; Zheng et al., 2008; Lin et al., 2015), and our data indicate that the selective localization of outposts to dendrites relies on the autoinhibition of axon-targeted kinesin-1.

## Results

### Survival and neuronal morphogenesis depend on E177 in β5-loop 8 of kinesin-1

**Figure 1.**
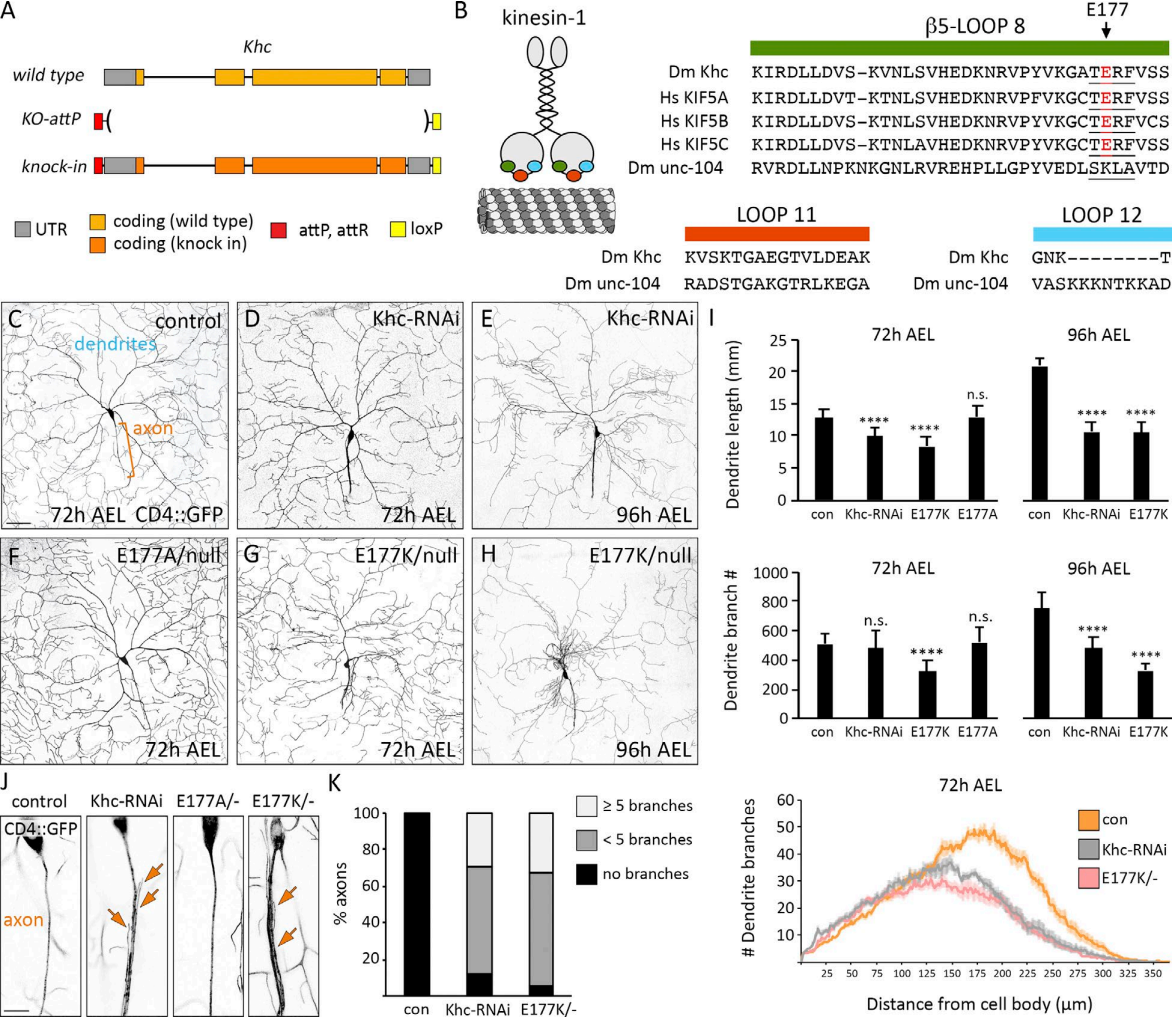
**Dendrite arborization is disrupted by charge-reversal mutagenesis of E177 in β5-loop 8 of Khc. (A)** Engineered *Khc* locus in which *Khc* is replaced by an *attP* site. **(B)** Cartoon of kinesin-1 highlighting β5-loop 8 (green), loop 11 (orange), and loop 12 (blue), which were replaced by equivalent loops from unc-104. β5-loop 8 is highly conserved between flies and humans. TERF sequence is underlined; arrow indicates Khc E177, also highlighted in red. **(C–H)** Representative images of control (con) and mutant neurons at 72 and 96 h AEL. Bar, 50 µm. **(I)** Quantification of dendrite length and branch number (mean ± SD) at 72 h AEL of 11 (*Khc^E177A/−^*), 13 (*Khc^E177K/−^*), and 16 (control, *Khc-RNAi*) neurons in at least three larvae and at 96 h AEL of 10 neurons (control, *Khc-RNAi*, *Khc^E177K/−^*) in at least three larvae. ****, P < 0.0001 relative to control and evaluated by one-way ANOVA and Tukey post hoc test (top and middle). Sholl analysis (bottom) of dendrite arbors at 72 h AEL (mean ± SEM). Critical radius and maximum branches reported in Table 2. **(J)** Zoomed-in view of proximal axons. The axons of *Khc^E177K/−^* and *Khc-RNAi*–expressing neurons frequently sprout ectopic branches (arrows). Bar, 10 µm. **(K)** Percentage of axons with ectopic branches in control, *Khc-RNAi*, and *Khc^E177K/−^* neurons (*n* = 18, all genotypes).

We initially set out to test whether kinesin-1 is stopped from carrying dendritic cargo into axons via the recognition of MT-based signals by its MTBD, which is comprised of three loops. To identify the loop or loops essential to transport, we took an in vivo structure–function approach and mutated endogenous kinesin-1 in fruit flies, which express one highly conserved kinesin-1, Kinesin heavy chain (Khc). Most of what is known about kinesin-1–mediated transport is derived from truncated motors. Thus, it is unclear which domain or domains are necessary for the full-length motor to mediate the selective localization of cargo in vivo. We took advantage of a fly strain we previously created that enables the rapid knock-in of *Khc* alleles via site-directed integration (Fig. 1 A; Winding et al., 2016). As *Khc* is an essential gene, we used viability to initially identify the loop or loops critical to Khc function.

We repeated loop-swap experiments that previously suggested β5-loop 8, loop 11, and loop 12 are integral to polarized transport (Nakata and Hirokawa, 2003; Konishi and Setou, 2009; Nakata et al., 2011; Huang and Banker, 2012). We substituted these highly conserved loops in Khc with equivalent loops from the structurally similar kinesin-3 (Table 1 and Fig. 1 B). Unlike kinesin-1, truncated kinesin-3 localizes to both axons and dendrites in mammalian neurons, suggesting it “reads” MT cues differently (Nakata and Hirokawa, 2003; Jacobson et al., 2006). Fruit flies express two kinesin-3 motors: unc-104 (also known as imac) and Khc-73. We swapped loops in Khc with equivalent loops from unc-104, which has essential roles in neuronal transport (Fig. 1 B; Pack-Chung et al., 2007; Barkus et al., 2008). Substituting loop 11 (*Khc^loop-11-swap^*) did not affect survival, indicating unc-104 loop 11 is able to functionally substitute for Khc loop 11 (Table 1). *Khc^loop-12-swap^* animals were homozygous viable; however, the *Khc^loop-12-swap^* mutation was lethal in trans to a null allele of *Khc*, suggesting *Khc^loop-12-swap^* is hypomorphic (Table 1). Swapping Khc β5-loop 8 with unc-104 β5-loop 8 resulted in lethality (Fig. 1 B and Table 1). This indicates that β5-loop 8 is essential to Khc function and that unc-104 β5-loop 8 cannot functionally substitute.

**Table 1.** Effect of kinesin-1 mutations on survival

**Khc allele**	**Description**	**Lethal stage**^a^	**Notes**
Control	Knock-in of wild-type kinesin-1 sequence	Not lethal	
Null	Q65-stop, protein null	Second instar	Also known as *Khc^27^* (Brendza et al., 1999)
Loop 12 swap	Loop 12 swapped for unc-104 loop 12	Not lethal	Lethal in trans to Khc null allele
Loop 11 swap	Loop 11 swapped for unc-104 loop 11	Not lethal	Not lethal in trans to Khc null allele
β5-loop 8 swap	β5-Loop 8 swapped for unc-104 β5-loop 8	Second instar	Lethal in trans to Khc null allele
SKLA	TERF in loop 8 swapped for equivalent unc-104 sequence	Third instar	
SQLA	TERF in loop 8 swapped for equivalent Khc-73 sequence	Not lethal	
E177K	Charge reversal mutation of E177 in β5-loop 8	Third instar	E177K knock-in recapitulates mutation in *Khc^19^* (Brendza et al., 1999)
E177R	Charge reversal mutation of E177 in β5-loop 8	Third instar	
E177A	Alanine substitution of E177 in β5-loop 8	Not lethal	Not lethal in trans to Khc null allele
S246F	Mutation in loop 11 that disrupts ATP hydrolysis	Pupae	S246F knock-in recapitulates mutation in *Khc^17^* (Brendza et al., 1999)
E164K	Mutation in β5-loop 8 that disrupts ATP hydrolysis	Third instar	Also known as *Khc^23^* (Brendza et al., 1999)
Δhinge2	Deletion of hinge 2 in stalk	Third instar	Disrupts autoinhibition, also known as KHCΔH2 (Barlan et al., 2013)
R947E	Charge-reversal mutation of R947 in the autoinhibitory IAK motif	Third instar	R947 and E177 form a salt bridge when the motor is autoinhibited (Kaan et al., 2011)
P945S	Mutation of P945S in the autoinhibitory IAK motif in the tail	Third instar	Also known as *Khc^22^* (Moua et al., 2011), P945S interacts with F179 when the motor is autoinhibited (Kaan et al., 2011)
K944E	Charge-reversal mutation of K944 in the autoinhibitory IAK motif	Third instar	
E177K, S246F	Charge reversal mutation of E177 combined with S246 mutation that decreases ATP hydrolysis	Pupae	
S181A, S182A	Alanine substitution of serines adjacent to TERF motif	Not lethal	Not lethal in trans to Khc null allele
S181D, S182D	Phosphomimetic mutation of serines adjacent to TERF motif	Not lethal	Lethal in trans to Khc null allele

a Stage of development when ≥75% of homozygous animals are dead.

β5-Loop 8 contains four amino acids (TERF) that were previously implicated in polarized transport (Konishi and Setou, 2009). The glutamate (E177) in this motif stabilizes the motor in an autoinhibited conformation, thus implicating this motif in both motor navigation and autoregulation (Kaan et al., 2011). We substituted the TERF sequence in Khc with the equivalent sequences from unc-104 and Khc-73. Strikingly, swapping Khc TERF with SKLA from unc-104 resulted in lethality, whereas mutating to the Khc-73 SQLA did not (Table 1). Notably, E177K had been previously uncovered in a genetic screen for lethal mutations in Khc (Brendza et al., 1999). Though mutating E177 to alanine (E177A) had no effect, we found that both charge-reversal mutations E177K and E177R caused lethality (Table 1).

We next determined how the Khc E177 mutations affected neuronal morphogenesis. The *Drosophila melanogaster* class IV dendritic arborization sensory neurons elaborate a highly branched dendritic arbor in the periphery and extend a single unbranched axon into the ventral nerve cord (VNC). Decreasing Khc levels reduced dendrite arborization and resulted in axons with minor ectopic branches (Fig. 1, C–E and I–K), consistent with research showing that Khc is essential for dendrite morphogenesis (Satoh et al., 2008). The viable *Khc^E177A^* mutation had no effect on neuron morphogenesis, but *Khc^E177K^* phenocopied *Khc-RNAi* (Table 2 and Fig. 1, F–K). The dendrite arborization phenotype of both the *Khc-RNAi* and *Khc^E177K^* neurons became more pronounced as the animals aged (Fig. 1, D, E, G, and H). Thus, the lethal E177 charge-reversal mutations disrupt neuron morphogenesis, indicating Khc-mediated transport is disrupted.

**Table 2.** Effects of Khc knockdown and mutations on dendrite arborization

Measurement	**Control**	**Khc-RNAi**	**E177K**	**S246F**	**S246F + E177K**
Critical radius	178 ± 16	148 ± 30**	141 ± 33**	169 ± 12 (NS)	154 ± 14*
Maximum branches	61 ± 6	49 ± 9***	39 ± 6****	57 ± 8 (NS)	61 ± 9 (NS)

Sholl analysis of dendritic arbors quantifies the number of dendrites that intersect concentric circles centered on the cell body. The radius of the circle with the maximum dendritic intersections is the critical radius and the intersections at this radius are the maximum number of branches. Mean ± SD for both measurements; n (neurons) = 14 control, 16 *Khc-RNAi*, 10 *Khc^E177K^*, 14 *Khc^S246F^*, 11 *Khc^S246F,E177K^*. *, P = 0.05–0.01; **, P = 0.01-0.001; ****, P < 0.0001 in comparison with control and evaluated by one-way ANOVA with Tukey post hoc test.

### The Khc E177K mutation disrupts the dendrite-specific localization of Golgi outposts and human transferrin receptor (hTfR)-positive vesicles

**Figure 2.**
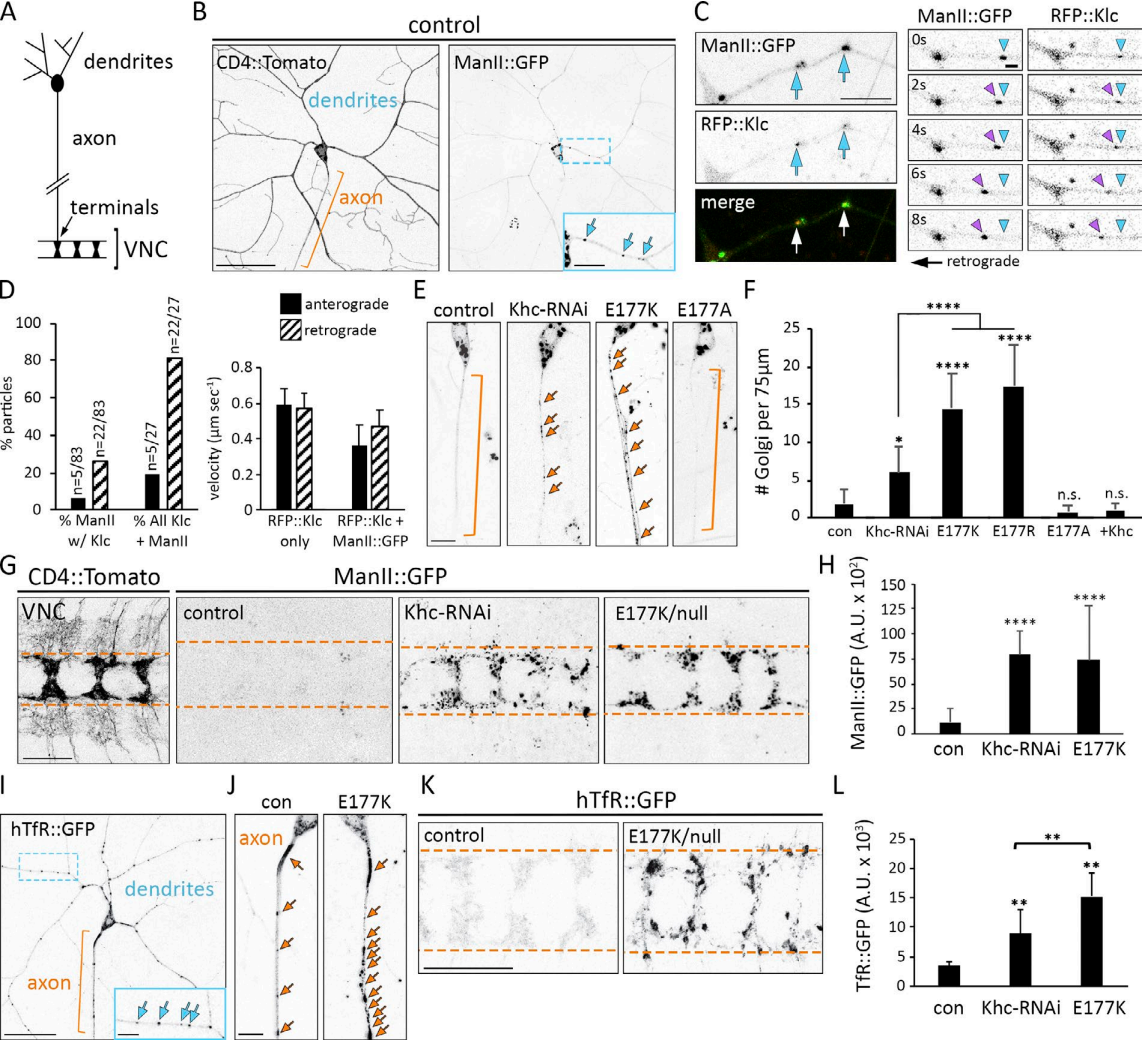
**Ectopic axonal localization of Golgi outposts and hTfR-positive vesicles results from Khc E177 charge-reversal mutations. (A)** Schematic of a class IV sensory neuron. In the VNC, multiple axons form a ladder-like pattern. **(B)** Golgi outposts labeled by ManII::GFP normally localize to dendrites. CD4::Tomato illuminates neuron morphology. Orange bracket indicates axon, and blue arrows indicate Golgi outposts. Bars: (main images) 50 µm; (inset) 20 µm. **(C)** Colocalization of Golgi outposts and Klc in dendrites. Individual video frames show a ManII- and Klc-positive punctum moving retrograde. Arrowheads mark start point (blue) and moving outpost (purple). Bars: (left) 10 µm; (right) 2 µm. **(D)** Quantification of RFP::Klc-positive Golgi outposts. 32% of moving ManII::GFP-positive outposts (27/83) colocalize with RFP::Klc. Of these RFP::Klc-positive outposts (*n* = 27), 81% move retrograde at speeds consistent with kinesin-mediated transport (*n* = 83 outposts and 75 RFP::Klc puncta in 24 neurons from 14 animals). RFP::Klc-only puncta may represent vesicles, which move faster than some organelles. **(E)** Golgi outposts mislocalize to axons in *Khc^E177K/−^* and *Khc-RNAi* neurons (unbranched axons were selected for clarity). Bar, 10 µm. Arrows indicate Golgi outposts. **(F)** Quantification of Golgi outposts (mean ± SD) in the proximal 75 µm of axons in 11 (*Khc^E177A/−^*), 12 (*Khc^E177K/−^*), 14 (*Khc^E177R/−^*), and 15 (control, *Khc-RNAi*, + Khc = *Khc^E177K/−^ ppk-Gal4 UAS-Khc::BFP*) neurons from at least five larvae. **(G)** Unlike control neurons, ManII::GFP accumulates in the axon terminals of *Khc^E177K/−^* and *Khc-RNAi* neurons. Bushy CD4::Tomato signal flanking axon terminals results from nonspecific transgene expression. Bar, 25 µm. Dashed orange lines demarcate axon terminal borders. **(H)** Quantification of ManII::GFP signal (mean ± SD) for 25 VNC segments from five larvae of each genotype. **(I)** hTfR::GFP normally localizes predominantly to dendrites. Bars: (main image) 50 µm; (inset), 20 µm. **(J)** Representative images of hTfR::GFP in control and mutant axons. Bar, 10 µm. **(K)** hTfR::GFP accumulates at the axon terminals of *Khc^E177K/−^* and *Khc-RNAi* neurons. Bar, 25 µm. Arrows indicate hTfR::GFP puncta (I and J), and dashed lines indicate axon terminal borders (K). **(L)** Quantification of hTfR::GFP signal (mean ± SD) in 25 VNC segments from five larvae of each genotype. *, P = 0.05–0.01; **, P = 0.01–0.001; ****, P < 0.0001 in comparison with control and evaluated by one-way ANOVA and Tukey post hoc test (F, H, and L).

The *Khc^E177K^* neurons resemble mutants in which dendritic transport is disrupted. Moreover, the ectopic axonal branching is characteristic of mutations that result in the axonal mislocalization of dendrite-specific Golgi outposts (Ye et al., 2007; Satoh et al., 2008; Zheng et al., 2008; Arthur et al., 2015). This led us to test whether Khc transports Golgi outposts and has a role in restricting them to dendrites. Kinesin-1 has been linked to Golgi membranes via its adapter kinesin light chain (Klc), which we found partially colocalizes with a subset of outposts in dendrites (Fig. 2, A–D; Marks et al., 1994; Gyoeva et al., 2000; Allan et al., 2002; Woźniak and Allan, 2006). A third of motile Golgi outposts colocalize with Klc, with most moving in a direction and at a velocity consistent with kinesin-mediated transport (Fig. 2 D). In control and *Khc^E177A^* neurons, Golgi outposts localized predominantly to dendrites (Fig. 2 B). In contrast, Golgi outposts mislocalized to axons in *Khc^E177K^*, *Khc^E177R^*, and *Khc-RNAi*–expressing neurons (Fig. 2, E and F). The mislocalization of outposts in *Khc^E177K^* neurons was rescued by the neuron-specific expression of wild-type Khc, indicating a cell autonomous requirement for Khc in outpost distribution. Mislocalized outposts were visible in both the proximal axon and axon terminals in the VNC (Fig. 2, G and H). These data indicate that Khc transports Golgi outposts and that the E177K and E177R mutations disrupt motor function to cause ectopic outpost localization.

To determine whether additional dendritic cargos are affected, we analyzed GFP-tagged hTfR. hTfR::GFP is a commonly used marker of dendritic vesicles that are transported, at least in part, by kinesin-1 and whose localization in fly neurons is Khc-dependent (Schmidt et al., 2009; Henthorn et al., 2011). In control neurons, hTfR::GFP was enriched in dendrites with some puncta in axons (Fig. 2, I and J). The *Khc^E177K^* mutation and *Khc-RNAi* resulted in an accumulation of hTfR::GFP in axons and axon terminals (Fig. 2, J–L). Although Golgi outposts and hTfR::GFP mislocalize to axons, we found no aberrant dendritic localization of three different axonal proteins (Fig. S1). Thus, Khc E177 has a critical role in restricting the localization of both Golgi outposts and hTfR::GFP-positive vesicles to dendrites.

### *Khc^E177K^* does not phenocopy mutations that disrupt kinesin-1 ATPase activity

**Figure 3.**
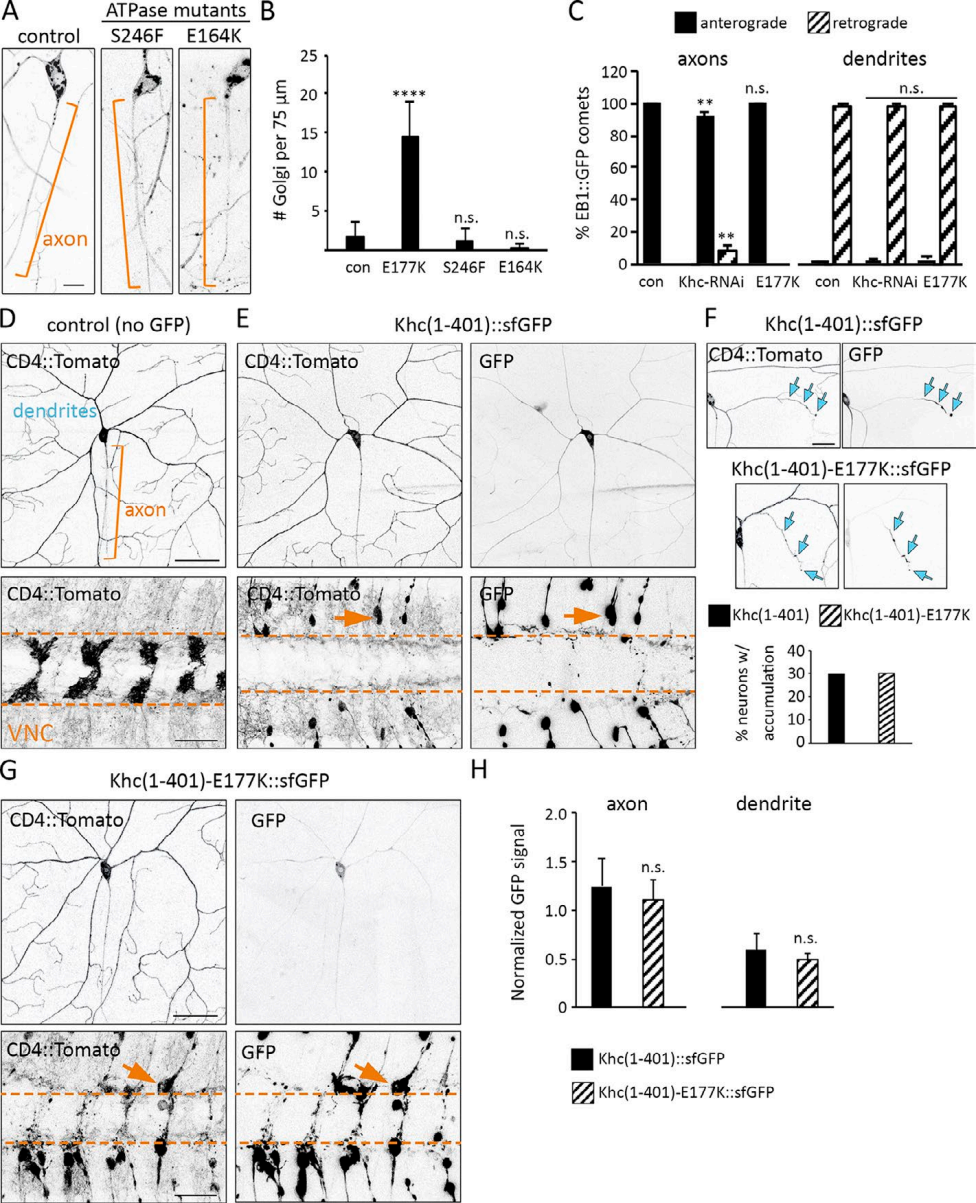
**The Khc E177K mutation does not phenocopy ATPase mutants, disrupt MT polarity, or affect the localization of truncated constitutively active Khc. (A)** Golgi outpost localization is not affected by the S246F and E164K mutations that disrupt ATPase activity. Brackets indicate axons, and arrows indicate Golgi outposts. Bar, 10 µm. **(B)** Quantification of Golgi outposts (mean ± SD) in the proximal 75 µm of axons in 12 (*Khc^E177K/−^*, *Khc^E164K/−^*), 13 (*Khc^S246F/−^*), and 15 (control) neurons from at least four larvae. **(C)** Quantification of EB1::GFP comets in the axons (left) and dendrites (right) of 10 (control, *Khc^E177K/−^*) and 12 (*Khc-RNAi*) neurons at 72 h AEL. **(D and E)** CD4::Tomato reveals the morphology of the proximal dendrites and axon (top) as well as axon terminals in the VNC (bottom). Khc(1–401)::sfGFP is in the cell body, diffuse in proximal axons and dendrites, and strongly accumulates at axon terminals, where it causes axon retraction (arrows). Bars: (top) 50 µm; (bottom) 25 µm. **(F)** Wild-type and mutant Khc(1–401)::sfGFP accumulate at one to three dendrite tips (arrows) in nearly a third of neurons. Bar, 25 µm. **(G)** Mutant Khc(1–401)-E177K::sfGFP behaves similarly to wild-type. Bars: (top) 50 µm; (bottom) 25 µm. Arrows indicate a retracting axon. **(H)** Quantification of the GFP signal (normalized to the raw Tomato signal) in the proximal axons and dendrites of 22 (Khc[1–401]-E177K::sfGFP) and 30 (Khc[1–401]::sfGFP) neurons. **, P = 0.01–0.001; ****, P < 0.0001 in comparison with control and evaluated by one-way ANOVA and Tukey post hoc test (B and C) or by a two-tailed Student’s *t* test (H).

The phenotypic similarity between *Khc^E177K^* and *Khc-RNAi* suggested that the mislocalization of Golgi outposts in the E177K mutant may be a result of decreased motor activity. However, the E177K mutation had no effect on the velocity or MT dwell time of truncated Khc in single-molecule motility assays (Fig. S2). Furthermore, Golgi outpost localization was unaffected by E164K or S246F mutations that alter ATPase activity and strongly affect the truncated motor’s landing rate and/or dwell time on MTs (Fig. 3, A and B; and Fig. S2; Brendza et al., 1999). Similarly, the E177A mutation did not affect outpost localization despite its reported effect on MT-stimulated ATPase activity and velocity (Woehlke et al., 1997). Thus, the E177K mutation is unlikely to disrupt Golgi outpost localization simply by diminishing Khc’s motility properties.

### Axonal MT polarity is perturbed by *Khc-RNAi* but not the E177K mutation

The normal localization of Golgi outposts in the Khc ATPase mutants raises the question of why outposts mislocalize to axons when Khc is absent. Decreasing kinesin-1 levels or function can disrupt MT organization in axons and dendrites (Yan et al., 2013; Lee et al., 2017). In *Khc-RNAi*–expressing neurons, axonal MT orientation was altered (Fig. 3 C), which may contribute to the ectopic axonal localization of outposts via dynein and/or another kinesin. MT organization in *Khc^E177K^* neurons was normal, indicating that a change in MT polarity does not underlie the mislocalization of Golgi outposts (Fig. 3 C). Ectopic Golgi outposts have been speculated to disrupt the polarity of axonal MTs because of their capacity to serve as platforms for MT nucleation (Ori-McKenney et al., 2012; Sanders and Kaverina, 2015). It is possible that the ectopic outposts in the *Khc^E177K^* axons may seed minus end–distal MTs, but that such MTs are cleared by mechanisms that normally maintain axonal MT polarity. Regardless, our data indicate that the ectopic outposts in *Khc^E177K^* axons are not a secondary consequence of a change in MT polarity.

### Localization of truncated constitutively active Khc is not affected by the E177K mutation

A motor’s ability to navigate in cells is typically assayed using truncated motors that lack cargo-binding and autoregulatory domains. Like the equivalent truncated mammalian kinesin-1 (Nakata and Hirokawa, 2003), truncated fly Khc(1–401)::superfolder GFP (sfGFP) was present at low levels in the proximal axon and dendrites but accumulated in axon terminals (Fig. 3, D and E). Notably, Khc(1–401)::sfGFP accumulated in one or two dendrite branches in 30% of neurons (Fig. 3 F), presumably because of the presence of some plus end–distal MTs in developing dendrite branches. The axon terminals of *Khc(1–401)::sfGFP* neurons had retracted, indicating that persistent expression of truncated Khc(1–401) may have a dominant-negative effect on axon growth (Seno et al., 2016). The mutant Khc(1–401)-E177K::sfGFP behaved similarly to the Khc(1–401)::sfGFP in all regards (Fig. 3, F–H). Thus, the E177K mutation does not noticeably disrupt the localization of the truncated constitutively active motor.

### The E177K mutation disrupts autoinhibition of kinesin-1

**Figure 4.**
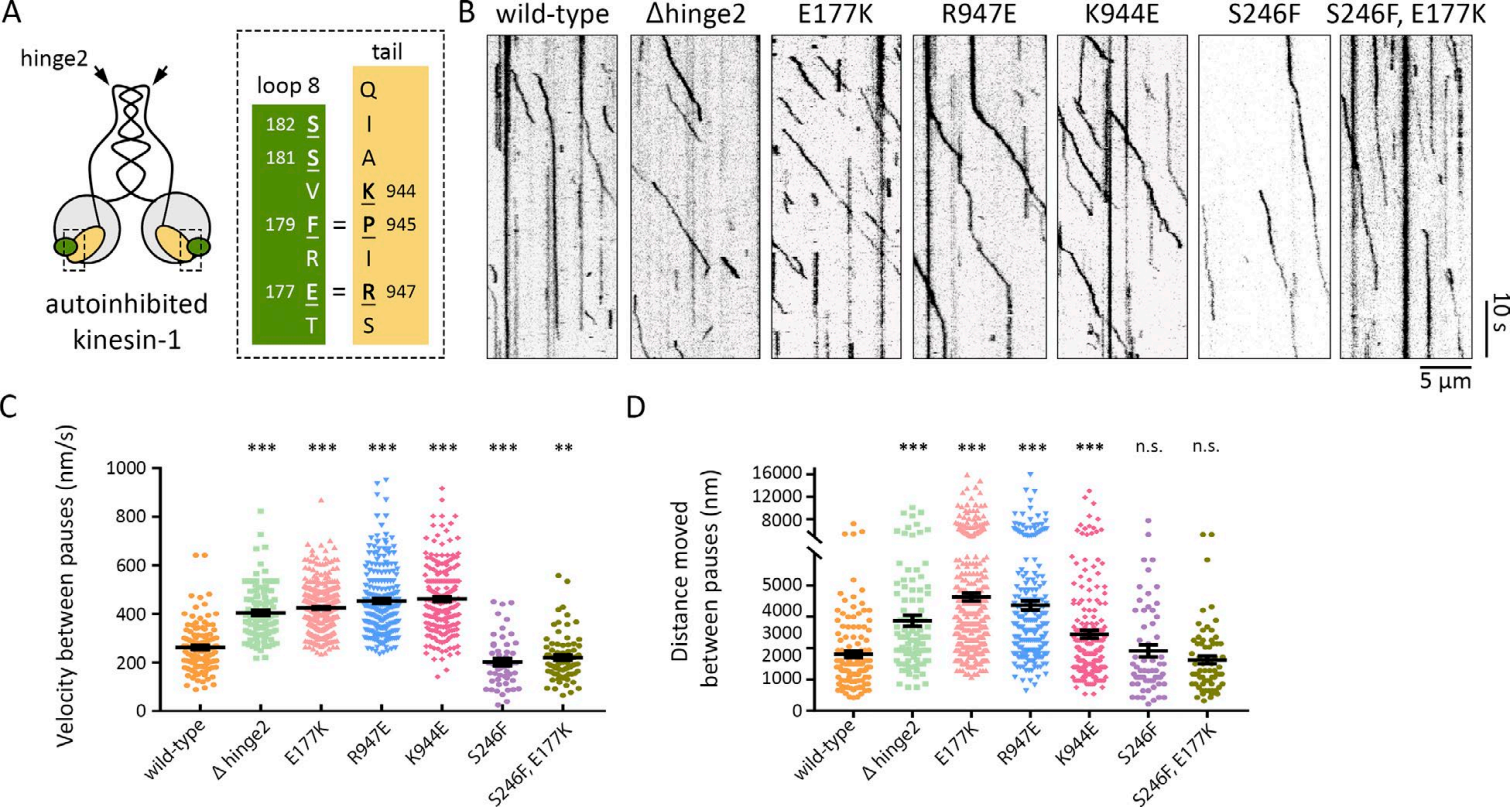
**The E177K mutation increases Khc motility in single-molecule motility assays. (A)** Kinesin-1 dimer in the folded autoinhibited conformation, which is stabilized by critical interactions (indicated by =) between residues in β5-loop 8 in the motor (green) and IAK motif in the tail (yellow). Mutated residues are underlined. **(B)** Kymographs showing single-molecule motility of full-length wild-type Khc and the indicated mutant versions. MTs are oriented plus end to the right. **(C and D)** Quantification of velocities (C) and distances (D) between pauses (mean ± SEM) for each population of motors. Motility events analyzed: wild-type, 121; Δhinge2, 105; E177K, 366; R947E, 225; K944E, 209; S246F, 52 (velocity) and 60 (run length); and E177K,S246F, 79 (velocity) and 66 (run length). **, P = 0.01–0.001; ***, P = 0.001–0.0001 in comparison with the wild-type motor and evaluated by a two-tailed Student's *t* test.

When kinesin-1 is in an autoinhibited conformation, E177 mediates an intramolecular salt bridge between the motor and tail (Fig. 4 A; Kaan et al., 2011). We tested whether the E177K mutation might alleviate autoinhibition by disrupting the motor-to-tail interaction. We first turned to an in vitro single-molecule approach. Wild-type or mutant full-length Khc motors tagged with mNeonGreen were added to flow cells containing MTs, and their motility was observed by total internal reflection fluorescence (TIRF) microscopy. As expected, very few motility events were observed for wild-type Khc, which is typically in an autoinhibited state in solution (Fig. 4 B). When motile, the motor typically moved only a short distance at slow speed before pausing or detaching (Fig. 4, B–D). As a positive control, we deleted the central hinge (Δhinge2) that enables the motor to fold on itself and adopt the autoinhibited state (Friedman and Vale, 1999). The Δhinge2 motors showed smooth movement along the MTs, with few pauses, and with speeds typical of an active kinesin-1 motor (Fig. 4, B–D), consistent with a previous study (Friedman and Vale, 1999). Motors with autoinhibition-disrupting mutations (E177K, R947E, and K944E) displayed motility that was characteristic of active kinesin-1 and was increased compared with the wild-type motor (Fig. 4, B–D; Hackney and Stock, 2000). The single-point mutant motors paused more than the Δhinge2 mutant motor, likely because these motors can still adopt the folded conformation, whereas the Δhinge2 mutant exists in an extended state. If the E177K mutation relieves autoinhibition, then the resultant activation should be ameliorated by decreasing ATPase activity. The double-mutant E177K, S246F motor behaved similarly to the single-mutant S246F and wild-type motors, indicating that reducing motor activity suppresses the effects of the E177K mutation (Fig. 4, B–D). These findings support the idea that E177 has a role in regulating kinesin-1 autoinhibition to control motor function.

### Disrupting autoinhibition perturbs Golgi outpost localization and dendrite morphogenesis

**Figure 5.**
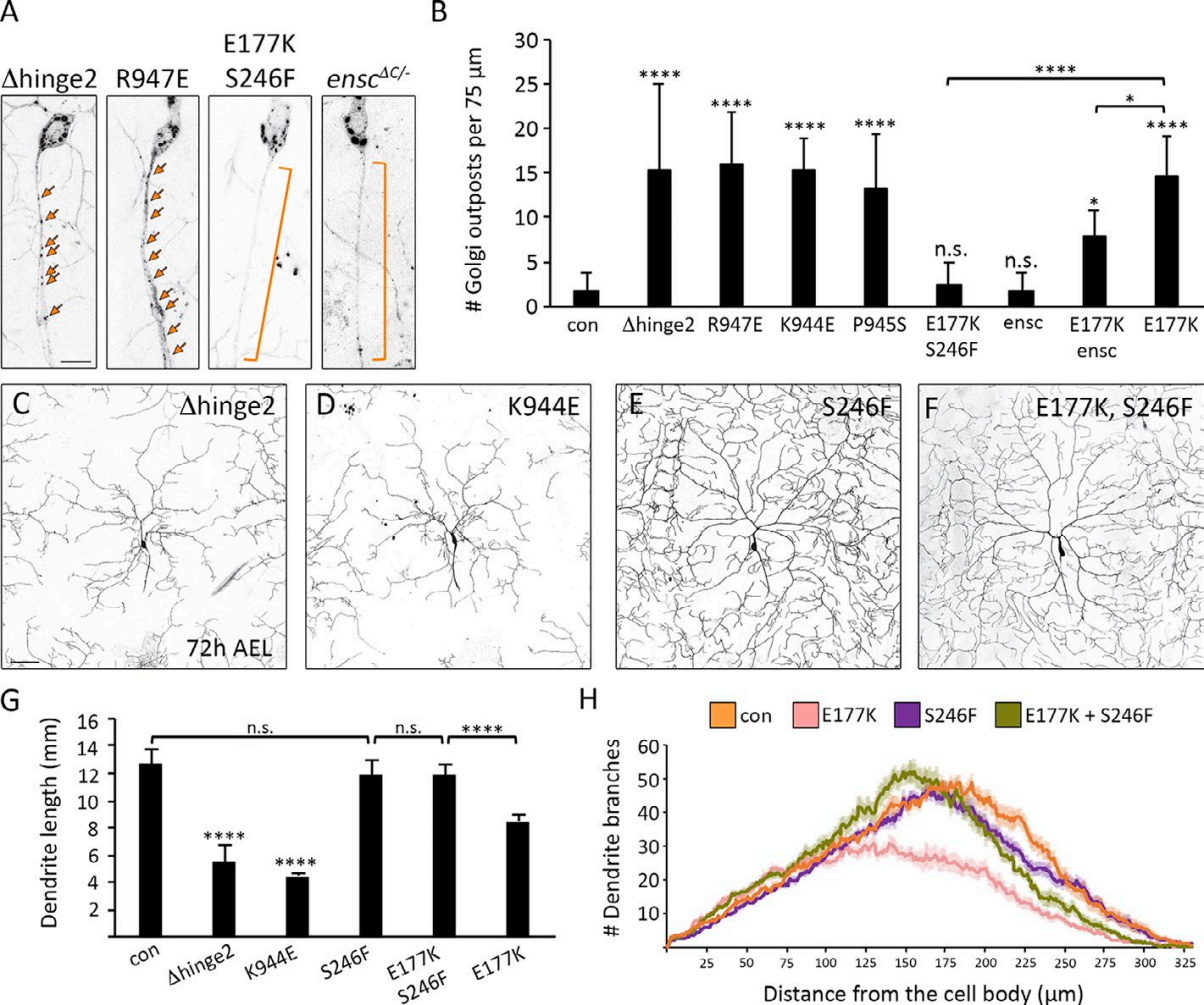
**Khc mutations that disrupt autoinhibition result in the axonal mislocalization of Golgi outposts and perturb dendrite arborization. (A and B)** Representative images and quantification of Golgi outposts in control and mutant neurons. Mutations that disrupt autoinhibition (Δhinge2, K944E, R947E, and P945S) result in the axonal mislocalization of Golgi outposts. Effects of the E177K mutation are rescued by the S246F mutation and the loss of ensconsin (*ensc^ΔC/−^*). Bracket indicates axon, and arrows indicate outposts. Bar, 10 µm. Quantification of Golgi outposts (mean ± SD) in the proximal 75 µm of axons in 12 (*Khc^Δhinge2/−^, Khc^E177K/−^*),13 (*Khc^R947E/−^*, *Khc^P945S/−^*), 14 (*Khc^K944E/−^*, *ensc^ΔC/−^*), and 15 (control, *Khc^E177K,S246F/−^*, double-mutant *Khc^E177K/−^*, *ensc^ΔC/−^*) neurons from at least five larvae. **(C–H)** Representative images (C–F) and quantification (G and H) of dendrite arbors in control and mutant neurons at 72 h AEL. Bar, 50 µm. Mutations that disrupt autoinhibition (Δhinge2, K944E) result in smaller dendritic arbors (C, D, and G). The S246F mutation does not affect dendrite morphology (E, G, and H) and rescues arbor reduction caused by the E177K mutation (F–H). Quantification (G) of total dendrite length (mean ± SD) and Sholl analysis (H) of branch distribution (mean ± SEM) in 13 (*Khc^E177K,S246F/−^*, *Khc^E177K/−^*), 15 (*Khc^Δhinge2/−^*, *Khc^K944E/−^*, *Khc^S246F/−^*), and 16 (control) neurons from at least four larvae (G). The critical radius and maximum branches determined by Sholl analysis are reported in Table 2. *, P = 0.05–0.01; ****, P < 0.0001; and n.s. relative to control and evaluated by one-way ANOVA and Tukey post hoc test (G and H).

Given the single-molecule evidence that E177K disrupts kinesin-1 autoinhibition, we next asked whether Golgi outpost localization would be affected by other mutations that disrupt autoinhibition. Indeed, all the mutations that alleviate kinesin-1 autoinhibition (Δhinge2, R947E, K944E, and P945S) resulted in the ectopic axonal localization of Golgi outposts (Fig. 5, A and B). And, like the E177K mutation, the Δhinge2 and K944E mutations significantly reduced dendrite arborization (Fig. 5, C–G). We also tested whether the S246F mutation would suppress the mislocalization of Golgi outposts caused by E177K. Neurons expressing the double-mutant E177K, S246F Khc did not have any mislocalized Golgi outposts in their axons and had relatively normal dendrite arbors (Table 2 and Fig. 5, A, B, and E–H). Reducing Khc activity reverses the effects of the E177K mutation, suggesting the E177K phenotypes result from increased motor activity. We further tested this idea by decreasing the levels of the kinesin-1 cofactor ensconsin in the E177K mutant neurons. Ensconsin (also known as MAP7) relieves autoinhibition in *Drosophila* neurons and embryos to promote kinesin-1 activation (Sung et al., 2008; Barlan et al., 2013). Loss of ensconsin by itself did not disrupt outpost localization, but loss of ensconsin in the *Khc^E177K^* neurons partially suppressed the mislocalization of Golgi outposts (Fig. 5, A and B). These data support the idea that a less inhibited, more active kinesin-1 ectopically transports Golgi outposts into axons.

We also tested whether Golgi outpost distribution was affected by the mutation of two conserved serines near E177 (S181 and S182) whose phosphorylation has been implicated in regulating kinesin-1 autoinhibition (Morfini et al., 2009). Golgi outpost localization and dendrite arborization were altered by phosphomimetic mutations but not alanine mutations (Fig. S3). This suggests that phosphorylation of these serines may favor the uninhibited active state, whereas dephosphorylation may allow the motor to switch between active and inhibited states.

### Loss of autoinhibition enriches Khc in axon terminals and reduces levels in dendrites

**Figure 6.**
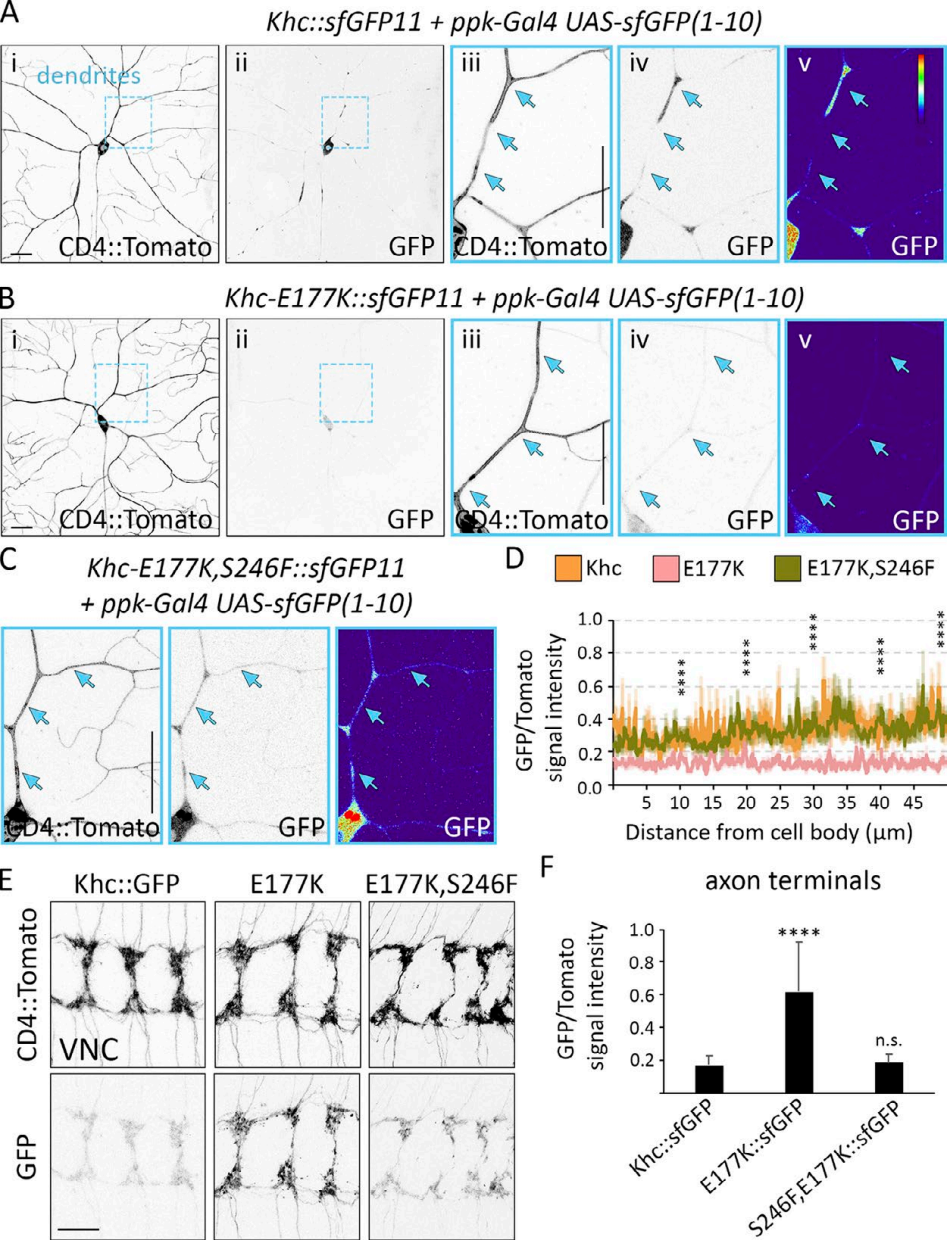
**Disrupting Khc autoinhibition results in axonal accumulation of the motor. (A–C)** Khc was tagged with sfGFP11, and Ppk-Gal4 drives the expression of UAS-sfGFP(1–10) to reconstitute sfGFP specifically in class IV sensory neurons. In contrast with wild-type Khc::sfGFP (A), Khc-E177K::sfGFP (B) is significantly reduced in cell bodies and dendrites. The double-mutant Khc-E177K,S246F::sfGFP localizes normally (C). **(D)** GFP signal intensity normalized to CD4::Tomato (mean ± SEM) along the proximal 50 µm of the brightest dendrite in 11 (Khc::sfGFP) and 12 (Khc-E177K::sfGFP, Khc-E177K,S246F::sfGFP) neurons from at least four larvae. The GFP/Tomato intensity ratios were compared at 10-µm intervals. Khc-E177K, S246F::sfGFP was not statistically distinct from Khc::sfGFP at any interval. **(E and F)** Representative images of sfGFP-tagged wild-type and mutant motors reveal that Khc-E177K::sfGFP accumulates at axon terminals (E). Motor localization is rescued by introducing the S246F mutation. (F) Quantification of the GFP/Tomato intensity ratios at axon terminals (mean ± SD) for 25 (Khc-E177K::sfGFP) and 29 (Khc::sfGFP, Khc-E177K,S246F::sfGFP) VNC segments in at least five larvae. ****, P < 0.0001 when compared with Khc::sfGFP and evaluated by one-way ANOVA and Tukey post hoc test (D and F). Bars, 25 μm. Arrows highlight the dendrite branch.

Because the E177K mutation enhances kinesin-1 activity, we reasoned the mutant motor might localize primarily to axons like the truncated constitutively active motor. The broad expression of *Khc* made it difficult to visualize GFP-tagged Khc in individual neurons, so we used split GFP (Cabantous et al., 2005; Kamiyama et al., 2016). We tagged endogenous Khc with sfGFP11 and restricted the expression of sfGFP(1–10) so that sfGFP would be reconstituted specifically in the class IV sensory neurons. Wild-type Khc localized to dendrites, axons, and axon terminals, whereas the E177K mutation resulted in a significant shift in Khc distribution from dendrites to axon terminals (Fig. 6, A, B, and D–F). The double-mutant E177K, S246F Khc localized normally to both dendrites and axons (Fig. 6, C–F). Thus, by lessening autoinhibition, the E177K mutation activates full-length Khc, depleting the motor from dendrites and resulting in its axonal enrichment.

### Reduced motility of Golgi outposts and hTfR-positive vesicles in E177K mutant dendrites

**Figure 7.**
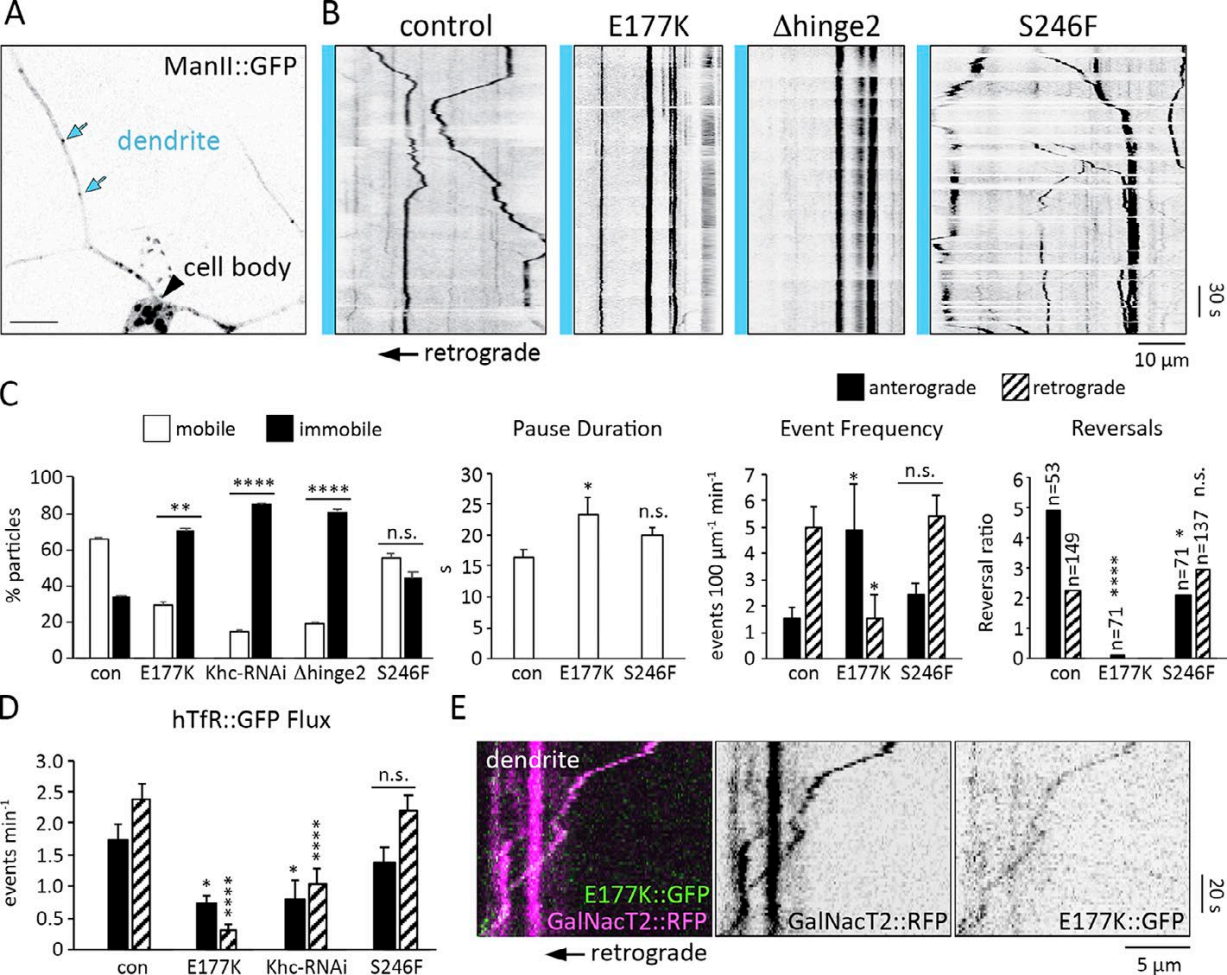
**Khc autoinhibition mutants decrease the motility of Golgi outposts and hTfR-positive vesicles in dendrites. (A)** Golgi outposts (blue arrows) in control dendrites. Bar, 10 µm. **(B)** Representative kymographs of Golgi outpost dynamics in control and mutant dendrites. Blue bar and arrow (below) indicate cell body is to the left. **(C)** Quantification of outpost motility, pause duration, event frequency, and reversals calculated for 7 (*Khc^E177K/−^*), 9 (control), and 10 (*Khc^S246F/−^*) neurons from at least five larvae (*Khc-RNAi* and *Khc^Δhinge2/−^* neurons had too few events for some calculations). The reversal ratio is the number of events that reversed direction after a pause relative to the number of events that did not; significant differences between the reversal ratios were determined via χ^2^ tests (n = number of events after a pause). **(D)** Quantification of hTfR::GFP flux in dendrites (mean ± SEM) in 9 (*Khc-RNAi* and *Khc^S246F/−^*), 11 (control), and 12 (*Khc^E177K/−^*) neurons from at least four larvae. *, P = 0.05–0.01; **, P = 0.01–0.001; ****, P < 0.0001; and n.s. relative to control and evaluated by one-way ANOVA and Tukey post hoc test (C and D). **(E)** Kymograph showing Khc-E177K::sfGFP (green) colocalizes with a Golgi outpost (GalNacT2::RFP, magenta) moving retrograde.

A significant decrease in dendritic Khc levels is likely to disrupt outpost motility. Consistent with this idea, mutations that alleviate autoinhibition reduced the fraction of motile outposts and increased their pause duration similar to *Khc-RNAi* (Fig. 7, A–C). The E177K mutation significantly reduced the frequency of retrograde events and the likelihood that outposts moving anterograde switched direction (Fig. 7 C). A significant decrease in anterograde reversals likely underlies the increased anterograde event frequency observed in the *Khc^E177K^* neurons (Fig. 7 C). Although pause duration increased, run length was normal in the mutants (Fig. S4 A). Golgi outposts were not the only cargo affected: the E177K mutation and *Khc-RNAi* also decreased hTfR::GFP flux, most strikingly retrograde flux (Figs. 7 D and S4 B). In contrast, the ATPase mutant S246F had little effect on Golgi outpost or hTfR::GFP motility (Fig. 7, B–D). This suggests that the Khc S246F mutant is sufficiently active to support transport in dendrites, which likely explains why dendrite growth is not affected by this mutation. Combined, our results show that depleting Khc from dendrites correlates with reduced outpost and hTfR::GFPmotility, with pronounced effects on retrograde transport.

Next, we attempted to visualize Golgi outposts being transported by the E177K mutant motor. We found it technically challenging to visualize Khc-E177K::sfGFP in mutant *Khc^E177K::sfGFP11/−^* neurons, but we did observe that Khc-E177K::sfGFP colocalized with retrogradely moving outposts in the dendrites of heterozygous *Khc^E177K::sfGFP11/+^* neurons (Fig. 7 E). Because one gene copy of the E177K mutation is not sufficient to drive outposts into axons, we could only observe colocalization in dendrites (only one Khc tail in a motor dimer is needed to inhibit activity). This colocalization is consistent with our model that the E177K mutant motor is able to actively transport Golgi outposts into axons.

### Khc interacts with dynein to regulate Golgi outpost localization

**Figure 8.**
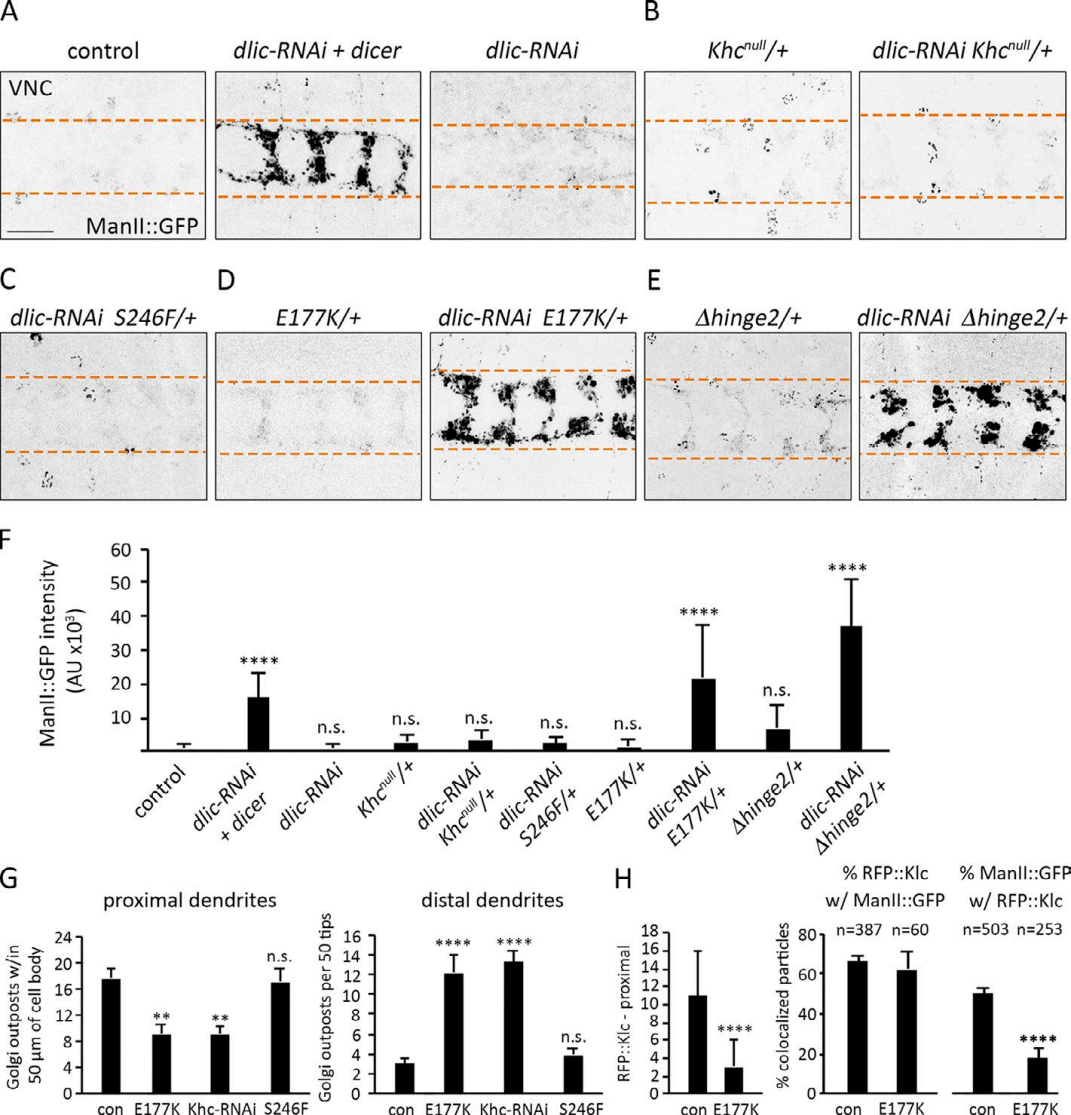
**Khc autoinhibition mutants interact with dynein to regulate Golgi outpost localization. (A–F)** Representative images (A–E) and quantification (F) of Golgi outposts at axon terminals in the VNCs of control and mutant animals. Bar, 25 µm. Dashed lines demarcate borders of axon terminals (A–E). One copy of *dlic-RNAi* alone does not cause Golgi outposts to mislocalize, but adding dicer results in a substantial accumulation of outposts at terminals (A and F). Reducing *Khc* dosage (*Khc^null/+^*) or activity (*Khc^S246^*^/+^) does not enhance *dlic-RNAi* (B, C, and F). *Khc^E177K/+^* or *Khc*^Δ^*^hinge2/+^* combined with *dlic-RNAi* results in ectopic Golgi outposts in terminals (D–F). Quantification of ManII::GFP signal intensity (mean ± SD) at axon terminals in 18 *(Khc^Δhinge2/+^*), 19 (*Khc^E177K/+^*, *Khc^null/+^*), 20 (*dlic-RNAi* + *dicer*, *dlic-RNAi* + *Khc^S246F/+^*), 24 (*dlic-RNAi*), and 25 (*dlic-RNAi* + *Khc^null/+^*, *dlic-RNAi* + *Khc^E177K/+^*, *dlic-RNAi* + *Khc^Δhinge2/+^*) VNC segments from at least four larvae (F). **(G)** Quantification of outpost distribution (mean ± SEM) in proximal (left) and distal (right) regions of dendrite arbors. Proximal: 14 (*Khc^E177K/−^*), 15 (control, *Khc-RNAi*, *Khc^S246F/−^*), and 16 (*Khc^E177K,S246F/−^*) neurons from at least six larvae. Distal: 20 (*Khc-RNAi*, *Khc^S246F/−^*, *Khc^E177K,S246F/−^*) and 24 (control, *Khc^E177K/−^*) neurons from at least six larvae. **(H)** Quantification of RFP::Klc puncta within 50 µm of the cell body (left) and colocalization of RFP::Klc and ManII::GFP (right) in dendrites of control and *Khc^E177K/−^* neurons. The number of RFP::Klc puncta is reduced, but the percentage of RFP::Klc puncta that colocalize with ManII::GFP is unchanged in *Khc^E177K/−^* neurons. **, P = 0.01–0.001; ****, P < 0.0001; and n.s. relative to control and evaluated by one-way ANOVA with Tukey post hoc test (F–H).

Golgi outposts are transported into dendrites by dynein (Ye et al., 2007; Zheng et al., 2008; Arthur et al., 2015), and the dendrite-specific localization of outposts may depend on a regulated balance of dynein and kinesin-1 activities. We used genetic interaction tests to determine whether disrupting the motor balance to favor Khc results in the ectopic axonal localization of outposts. We used RNAi targeting the dynein light intermediate chain (dlic) subunit (*dlic-RNAi*) to reduce dynein function. A strong decrease in dynein function (*dlic-RNAi* and *dicer*) resulted in the ectopic accumulation of outposts at axon terminals, consistent with the idea that a shift favoring kinesin enables the motor to pull outposts into axons (Fig. 8 A). A weak dynein loss of function (*dlic-RNAi* alone) did not affect outpost distribution, providing a paradigm to test for genetic interactions (Fig. 8 A). In the weak-dynein loss-of-function background, Golgi outpost distribution was not affected by reducing Khc activity by either removing one copy of *Khc* or decreasing ATPase activity (Fig. 8, B, C, and F). This is consistent with the notion that attenuating the activity of both dynein and kinesin-1 simultaneously maintains a motor balance that restricts Golgi outposts to dendrites.

Next, we tested whether reducing Khc autoinhibition in the weak-dynein loss-of-function background would disrupt the motor balance and Golgi outpost localization. One copy of *Khc^E177K^* in a control background had no effect on outpost distribution (Fig. 8, D and F). However, one copy of *Khc^E177K^* coupled with a mild reduction of dynein function resulted in the mislocalization of Golgi outposts to axon terminals (Fig. 8, D and F). Similarly, one copy of *Khc^Δhinge2^* combined with *dlic-RNAi* resulted in ectopic Golgi outposts at axon terminals (Fig. 8, E and F). These results support the idea that relieving Khc autoinhibition tips the dynein-kinesin balance toward kinesin-1 and biases transport into axons.

If a balance of dynein and kinesin-1 regulates Golgi outpost distribution, then reducing kinesin-1 levels in dendrites should cause a distal shift of outposts in the arbor. Consistent with this idea, knocking down Khc reduced the number of outposts proximally and increased outposts distally (Fig. 8 G). The E177K mutation, which reduces Khc levels in dendrites, resulted in a similar shift in outpost localization. Although there are more Golgi outposts distally, we did not observe a corresponding shift in dendrite branches such as has been reported for mutations that alter outpost distribution or the dynein-kinesin balance (Ye et al., 2007; Satoh et al., 2008; Zheng et al., 2008; Arthur et al., 2015; Taylor et al., 2015). It is possible that kinesin-1 acts downstream of Golgi outposts to promote dendrite branching, or that loss of Khc disrupts the transport of additional factors needed for branching. Of the Golgi outposts remaining in the proximal dendrites of the E177K mutants, significantly fewer colocalized with RFP::Klc, and overall fewer RFP::Klc puncta were present, paralleling the reduction in Khc levels (Fig. 8 H). Thus, lessening Khc autoinhibition results in Khc, its cofactor Klc, and outposts moving out of dendrites and causes a distal shift of remaining outposts, likely because of an unopposed dynein motor.

## Discussion

Neuronal function relies on the accurate delivery of proteins, vesicles, and organelles to axons and dendrites by molecular motors. Our in vivo structure–function analysis of endogenous Khc, combined with in vitro single-molecule experiments, indicate that regulated autoinhibition of kinesin-1 is necessary for the polarized distribution of Golgi outposts and hTfR-positive vesicles to dendrites. Previous studies suggested that autoinhibition may be dispensable for some Khc-mediated activities, whereas the autoinhibitory IAK tail motif is critical to axonal transport (Moua et al., 2011; Williams et al., 2014). Our results indicate that kinesin-1 autoinhibition has a previously unrecognized function in preventing the motor from carrying Golgi outposts into axons. Relieving autoinhibition enhances Khc activity and drives the cargo-bound motor into axons, depleting dendrites of Khc and perturbing dendrite arborization. Disrupting autoinhibition generates phenotypes that resemble kinesin-1 loss of function. However, these effects can be reversed by decreasing ATPase activity, which strongly indicates these phenotypes are a result of a gain in motor activity, not a loss. It is likely that the depletion of the motor from a particular compartment, such as dendrites, contributes to the loss-of-function phenotypes observed when kinesin-1 autoinhibition is disrupted. Thus, tight regulation of kinesin-1 activity is critical to the polarized localization of dendritic cargo, the motor itself, and neuronal morphogenesis.

We had initially sought to determine whether kinesin-1 is stopped from carrying dendritic cargo into axons by reading out MT-based signals such as MT posttranslational modifications, MT-associated proteins, and/or GTP-tubulin subunits (Bentley and Banker, 2016). Mutagenesis of the three loops in the kinesin-1 MTBD revealed that loop 12 and β5-loop 8 are required for normal kinesin-1 function. Swapping loop 12 may decrease animal survival because of the unique K-loop in loop 12 of kinesin-3 increasing the on-rate of the motor onto MTs (Soppina and Verhey, 2014). Swapping β5-loop 8 had a more severe effect on survival. Within β5-loop 8, we found that an essential glutamate (E177) is highly sensitive to the nature of substitution. Charge-removal substitutions (E177A and E177Q) had no effect on survival; however, charge-reversal substitutions (E177K or E177R) were lethal and altered dendritic trafficking. Based on previous studies, we expected that the underlying mechanism would be an alteration of either kinesin-1 responsiveness to MT-based signals such as MT detyrosination (Konishi and Setou, 2009) or autoinhibition (Kaan et al., 2011). We found that the E177K mutation increases motor activity in vitro, causes the full-length motor to localize to axon terminals, and can be combined with dynein loss of function to drive outposts into axons. Additional autoinhibition-disrupting mutations in the Khc tail domain also result in the axonal mislocalization of Golgi outposts. We thus favor the idea that the loss of Khc inhibition is responsible for altered dendritic trafficking in E177K mutants. Although we cannot rule out the possibility that E177 is involved in track selection, structural studies indicate the TERF sequence may not be positioned to directly respond to MT modifications (Gigant et al., 2013; Atherton et al., 2014). Combined, our data suggest that E177 regulates motor activity through autoinhibition, which we propose functions as a brake on the axonal localization of the motor when it is carrying dendritic cargo. It will be interesting to test how the E177K mutation and loss of autoinhibition affect the localization and function of full-length kinesin-1 in mammalian neurons, whose dendritic MTs have a more mixed polarity than fly.

Our autoinhibition model does not exclude other models of polarized transport, and indeed we believe that the regulated autoinhibition of kinesin-1 acts in concert with other mechanisms to control cargo distribution. Furthermore, models that take into account potential indirect effects of kinesin-1 autoinhibition on outpost localization are also possible. Kinesin-1 has been previously implicated in dynein localization, and some aspects of dynein activity may depend on kinesin-1 (Satoh et al., 2008; Hancock, 2014; Twelvetrees et al., 2016). Interfering with kinesin-1 autoinhibition might disrupt the localization and/or function of dynein and thereby enable a kinesin to pull outposts into axons. Although this could be kinesin-1 itself, it is also possible that an additional kinesin is involved in transporting outposts. The best candidates for such another kinesin would be kinesin-2 or kinesin-3, which cotransport some cargo with kinesin-1 (Hendricks et al., 2010; Guardia et al., 2016; Ruane et al., 2016; Gumy et al., 2017; Kulkarni et al., 2017; Lim et al., 2017). Cotransport with another kinesin could also explain why knocking down Khc levels (Khc-RNAi) but not Khc activity (S246F and E164K) causes the Golgi outposts to mislocalize to axons. The loss of Khc might allow another kinesin that is regulated differently (or less precisely) than kinesin-1 to ectopically propel outposts into axons.

Our study raises the question of how kinesin-1 autoinhibition is spatially regulated. It is likely that autoinhibition of kinesin-1 attached to dendritic cargo would be triggered in the cell body or proximal axon, where dendritic cargo is turned around (Edwards et al., 2013; Rolls and Jegla, 2015; Bentley and Banker, 2016). In the proximal axon of fly sensory neurons, Golgi outposts colocalize with the dynein cofactor NudE, which likely functions with dynein to localize outposts to dendrites (Ye et al., 2007; Zheng et al., 2008; Arthur et al., 2015; Lin et al., 2015). The mammalian NudE orthologue Ndel1 is proposed to “catch” dendritic cargos in the proximal axon and prime them for dynein-mediated transport into dendrites (Kuijpers et al., 2016; Klinman et al., 2017). We found that simultaneously decreasing dynein activity and kinesin-1 autoinhibition resulted in ectopic axonal Golgi outposts. This suggests that the selective transport of Golgi outposts to dendrites relies on coordinating the activation of a dendrite-targeted motor (dynein) and the inhibition of a motor that targets axons (kinesin). A key future goal is to determine how kinesin-1 autoinhibition is spatially regulated to mediate the proper distribution of cargo to axons or dendrites to maintain neuronal polarity.

## Materials and methods

### Molecular cloning

#### *Khc* knock-in alleles

The *Khc* knock-in alleles were generated using the fly strain *Khc^KO-attP^*, in which *Khc* is knocked out and replaced with an *attP* site for rapid site-directed integration of new alleles (Winding et al., 2016). To knock in new Khc alleles, an integration plasmid (*pGE-attB-GMR*; Huang et al., 2009) containing the new allele was injected into *Khc^KO-attP^* embryos and integrated via ϕC31-mediated recombination. The following mutations were introduced by PCR-based mutagenesis using Q5 High-fidelity polymerase (New England Biolabs, Inc.): loop-11 swap, loop-12 swap, SKLA, SQLA, E177A, E177K, E177R, Δhinge2, R947E, K944E, S246F, E177K + S246F, S181D + S182D, and S181A + S182A. To make the β5-loop 8 swap mutant, the *unc-104* sequence that encodes β5-loop 8 was ordered as a gBlock (Integrated DNA Technologies) and the final construct was created via Gibson Assembly (New England Biolabs, Inc.). The amino acid sequences that were swapped are as follows: Khc β5-loop 8 (KIRDLLDVSKVNLSVHEDKNRVPYVKGATERFVSS) was substituted with RVRDLLNPKNKGNLRVREHPLLGPYVEDLSKLAVTD from unc-104; Khc loop 11 (KVSKTGAEGTVLDEAK) was substituted with RADSTGAKGTRLKEGA from unc-104; and Khc loop 12 (GNKT) was substituted with VASKKKNTKKAD from unc-104. To make Khc::sfGFP11, the sfGFP11 peptide (multimerized 7×) was added to the C-terminal end of Khc with a GGSGG linker between Khc and sfGFP11 and successive sfGFP11 peptides. All knock-in plasmids were fully sequenced before injection (BestGene Inc.). The oligonucleotides and gene fragments used to make these and all other constructs are listed in Table S1.

#### Transgenic flies

*UAS-Khc(1–401)::sfGFP* was assembled by first using HiFi DNA assembly (New England Biolabs, Inc.) to piece together a sfGFP-encoding gene block (Integrated DNA Technologies) and *pIHEU-MCS* plasmid (plasmid 58375; Addgene) to create *sfGFP-pIHEU*. The *pTTSTOP* plasmid containing truncated Khc, residues 1–401 (plasmid 41751; Addgene; Thoresen and Gelles, 2008) was digested, subcloned, and then ligated into *sfGFP-pIHEU* to create *UAS-Khc(1–401)::sfGFP*. PCR-based mutagenesis of *UAS-Khc(1–401)::sfGFP* using Q5 high-fidelity polymerase (New England Biolabs, Inc.) and Gibson assembly were used to create *UAS-Khc(1–401)-E177K::sfGFP*. The plasmids were injected into strain *P{CaryP}attP40* (BestGene Inc.). *Pickpocket* (*Ppk*)-*ManII::GFP*, which expresses Mannosidase II (ManII)::GFP (Ye et al., 2007) under the control of the *ppk* enhancer, was created by PCR-amplifying *ManII::GFP* and cloning it into *pDEST-APPHIH*, which contains a 1-kb *ppk* enhancer fragment (provided by C. Han, Cornell University, Ithaca, NY). *Ppk-ManII::GFP* was injected into strain *PBac{yellow[+]-attP-3B}VK00037* (Bloomington Drosophila Stock Center [BDSC] stock no. 9752; BestGene Inc.). *UAS-sfGFP(1–10)*, which was made by cloning sfGFP(1–10) into *pACUH* (plasmid 58374; Addgene; Kamiyama et al., 2016), was injected into strain *PBac{y[+]-attP-3B}VK00027* (Rainbow Transgenic Flies, Inc.).

### Fly genetics

The Khc alleles *Khc^27^* (null allele), *Khc^22^* (amino acid replacement P945S), and *Khc^23^* (amino acid replacement E164K) were provided by W. Saxton, University of California, Santa Cruz, Santa Cruz, CA (Brendza et al., 1999). UAS-Khc::BFP was provided by V. Gelfand (Northwestern University, Chicago, IL; Winding et al., 2016). The following fly strains were obtained from the BDSC and the Vienna *Drosophila* Resource Center: *UAS-hTfR::GFP* (BDSC stock no. 36858); *ppk-CD4::Tomato* (BDSC stock no. 35845); *ppk-CD4::GFP* (BDSC stock no. 35843); *ppk-Gal4* (BDSC stock nos. 32078 and 32079); *ensconsin* alleles, including *ensc^ΔC^*, which lacks the majority of coding exons (Sung et al., 2008; BDSC stock no. 51318), and *Df(3L)ensc^Δ3277^*, a deficiency that removes *ensconsin* (BDSC stock no. 51319); *UAS-Khc-RNAi* (BDSC stock no. 35770); *UAS-dicer2* (BDSC stock no. 24650); and *UAS-dlic-RNAi* (stock no. 41686; Vienna Drosophila Stock Center). All transgenic markers (e.g., *ppk-ManII::GFP*) were used as a single copy. Khc mutants were analyzed in trans to the null allele *Khc^2^*^7^ unless otherwise noted.

### Image capture and analysis

All neuron imaging was performed on an SP5 confocal microscope (Leica Microsystems). The dorsal class IV dendritic arborization neurons (ddaC) in the abdominal segments of control and mutant larvae were imaged in live animals. Live larvae were mounted on slides in a solution of 50% PBS and 50% glycerol and immobilized by pressing a coverslip mounted on top of two lines of vacuum grease spacers flanking the animal. All images obtained from *Drosophila* larvae were acquired at 25°C from a hybrid detector (Leica Microsystems) using the Leica Application Suite software.

To quantify the number of ManII-positive Golgi outposts in axons, third-instar larvae expressing ManII::GFP and CD4::Tomato in ddaC neurons were imaged at a resolution of 1,024 × 1,024 pixels using a 40 × 1.3 NA oil-immersion objective. Z stacks 10–20 µm thick (1.5 µm per z step) were maximum projected, and the number of ManII::GFP puncta was quantified using the FIJI particle analysis function (ImageJ; National Institutes of Health). The ManII::GFP signal in black-and-white images was inverted and subject to threshold with a cut-off gray value of 170. ManII::GFP-positive puncta between 0.15–15.00 µm in diameter within 75 µm of the cell body (axon) or a 50-µm radius of the cell body (proximal dendrites) were counted. To calculate distal Golgi outposts per dendrite branch, a box of 125 × 250 µm was drawn around the distal tips closest to the dorsal midline. This area was inverted and subject to threshold with a cut-off gray value of 140. ManII::GFP-positive puncta between 0.10–15.00 µm in diameter were counted. The number of tips in the same region was counted using the CD4::Tomato channel.

Golgi outpost colocalization with Klc was determined by simultaneous imaging of ManII::GFP and RFP::Klc for 2–5 min in ddaC dendrites of third instar larvae. Movies were captured at a rate of 0.667 frames/s and a resolution of 1,024 × 512 pixels using a 40 × 1.3 NA oil-immersion objective. Videos were stabilized using the FIJI Image Stabilizer plugin, and kymographs were generated using ImageJ. Kymographs were used to determine colocalization between Golgi outposts and Klc as well as to calculate the directionality and velocity.

Golgi outpost motility was quantified by imaging ManII::GFP puncta for 4–6 min in one or two ddaC neurons per larva at 72 h after egg laying (AEL). Videos were captured at a rate of 0.895 frames/s and a resolution of 1,024 × 512 pixels using a 40 × 1.3 NA oil-immersion objective. ManII::GFP puncta within 100 µm of the cell body in primary and secondary dendrites were analyzed. Videos were stabilized using the FIJI Image Stabilizer plugin, and kymographs were generated using MetaMorph (Molecular Devices). Position and time data from MetaMorph were exported to Excel to quantify directionality. Puncta that traveled consistently in one direction were classified as anterograde or retrograde, whereas those that switched direction were classified as bidirectional. Puncta that did not move during the video were classified as paused. Kymographs were further in analyzed MetaMorph to quantify the number, frequency, velocity, run length, and pause duration of anterograde and retrograde events. The event frequency was calculated only for videos with motile outposts. Particles that moved, paused for at least three frames (2.62 s), and then resumed movement were used to calculate direction changes and determine the frequency of direction switching.

hTfR::GFP was imaged using similar conditions and protocols as described for Golgi outposts. In brief, hTfR::GFP puncta were imaged for 4–6 min in one or two ddaC neurons per larva at 72 h AEL. Movies were captured at a rate of 0.895 frames/s and a resolution of 1,024 × 512 pixels using a 40 × 1.3 NA oil-immersion objective. GFP puncta within 100 µm of the cell body in primary and secondary dendrites were analyzed. Videos were stabilized using the FIJI Image Stabilizer plugin and kymographs were generated using MetaMorph. Position and time data from MetaMorph were exported to Excel to quantify directionality. Robust hTfR::GFP motility and frequent puncta crossings made single-particle tracking impractical. Flux was calculated based on the number of particles that crossed the midpoint of a 75-µm-long kymograph per minute. Particles were classified as anterograde or retrograde based on their directionality as they crossed the midpoint of the kymograph.

Dendrite length, branch points, and Sholl analysis were quantified by imaging two to four ddaC neurons from segments A3–A6 in larvae at 72 and 96 h AEL at a resolution of 2,048 × 2,048 pixels using a 20 × 0.7 NA oil immersion objective. Z stacks 10–15-µm thick (1 µm per z step) were analyzed using the FilamentTracer module in the Imaris software program (Bitplane) to obtain dendrite length, branch point number, and Sholl crossings. Neurons were analyzed individually by masking signals from neighboring neurons. Data were exported to Excel for statistical analysis. Axon branching was assessed for late third instar larvae by counting branch points in individual axons. Axons were split into three categories: unbranched, fewer than five branches, or five and more branches.

Truncated Khc(1–401)::sfGFP localization was quantified by imaging two to four ddaC neurons from segments A3–A5 in third instar larvae at a resolution of 1,024 × 1,024 pixels using a 40 × 1.3 NA oil immersion objective. Z stacks 10–15-µm thick (1 µm per z step) were analyzed using FIJI software to determine the background-subtracted GFP intensity and Tomato signal in the proximal dendrites and axons 50 µm from the cell body. The ratio of GFP/Tomato was calculated for each neurite analyzed.

MT polarity was analyzed by imaging EB1::GFP puncta within 100 µm of the cell body for 4–6 min in one or two ddaC neurons per larva at 72 h AEL. Videos were captured at a rate of 0.895 frames/s and a resolution of 1,024 × 512 pixels using a 40 × 1.3 NA oil-immersion objective. Videos were stabilized using the FIJI Image Stabilizer plugin, and kymographs were generated using MetaMorph. Position and time data from MetaMorph were exported to Excel to quantify directionality.

To image ManII::GFP, Khc(1–401)::sfGFP, and hTfR::GFP in the VNC, brains from third instar larvae were dissected in 1× PBS, fixed in 4% paraformaldehyde for 20 min, washed with 1× PBS, and mounted on slides in a solution of elvanol between vacuum grease spacers. Fixed VNCs were imaged using a 40 × 1.3 NA oil-immersion objective. Z stacks 20–30 µm thick (1 µm per z step) were analyzed using FIJI. The background-subtracted, integrated fluorescence intensity within a 20 × 50-µm box drawn around commissures was quantified in four to five terminals per larva (segments A2–A6).

The fluorescent signal of reconstituted sfGFP in axon terminals expressing Khc::sfGFP11 and sfGFP(1–10) was quantified by determining the background-subtracted, integrated fluorescence intensity of both Khc::sfGFP and CD4::Tomato within a 20 × 50-µm box drawn around commissures (segments A2–A6). The ratio of GFP/Tomato signal was determined for each commissure analyzed. The Khc::sfGFP/Tomato signal in dendrites was similarly quantified in FIJI by drawing a 50-µm-long line starting from the cell body and tracing the primary dendrite with the brightest GFP signal. The mean intensity over 10-µm segments was statistically analyzed.

### Antibody staining

To image bruchpilot, cysteine string protein, and synapsin signal in sensory neurons, third instar larvae were dissected in 1× PBS, fixed in 4% paraformaldehyde for 45 min, washed with 1× PBS, permeabilized in 1× PBS, 0.3% Triton X-100 (PBSTx) for 20 min, washed with PBSTx, quenched in 50 mM NH_4_Cl for 10 min, washed with PBSTx, and incubated in block for at least 1 h. Larval fillets were incubated in primary antibody overnight, washed (3 × 30 min in PBSTx), and then incubated in fluorescent secondary antibodies overnight. Fillets were then washed in PBSTx (3 × 30 min) and mounted in a solution of elvanol. Fixed larvae were imaged using a 40 × 1.3 NA oil-immersion objective. Z stacks 20–30 µm thick (1 µm per z step) were analyzed using FIJI. Mouse antibodies that recognize bruchpilot (nc82), cysteine string protein (DCSP-2 6D6), and synapsin (3C11) were obtained from the Developmental Studies Hybridoma Bank.

### S2 cell lysates

*Drosophila* S2 cells were cultured in Schneider’s *Drosophila* medium (Gibco) supplemented with 10% (vol/vol) FBS (HyClone) at 25°C. Constructs expressing *Drosophila* Khc tagged with mNeonGreen at the C terminus in the pMT vector were transfected into cells using Cellfectin II (Invitrogen), and protein expression was induced by adding 1 mM CuSO_4_ to the medium 4–5 h posttransfection. The cells were harvested after 48 h.

Motor-expressing S2 cells were harvested and centrifuged at low speed at 4°C. The cell pellet was washed once with PBS and resuspended in ice-cold lysis buffer (25 mM Hepes/KOH, pH 7.4, 115 mM potassium acetate, 5 mM sodium acetate, 5 mM MgCl_2_, 0.5 mM EGTA, and 1% vol/vol Triton X-100) freshly supplemented with 1 mM ATP, 1 mM PMSF, protease inhibitors cocktail (P8340; Sigma-Aldrich), 10% glycerol, and 1 mM DTT. After clarifying the lysate by centrifugation at 16,000 *g* at 4°C, aliquots were snap frozen in liquid nitrogen and stored at −80°C until further use. The relative amount of motor across lysates was calculated by a dot blot in which serial dilutions of each S2 lysate were spotted onto a nitrocellulose membrane (GE Healthcare). The membrane was air-dried and immunoblotted with a polyclonal antibody to kinesin-1 generated against the motor domain peptide (KLSGKLYLVDLAGSEKVSKTGAEG; Verhey et al., 1998). The amount of Khc in each spot was quantified (ImageJ), and spots within the linear regimen were used to normalize motor levels for addition to single-molecule motility assays.

### Single-molecule motility assays

All single-molecule assays were performed at room temperature in a flow cell (∼10 µl volume) prepared by attaching a clean no. 1.5 coverslip to a glass slide with double-sided tape. HiLyte-647–labeled MTs were polymerized from a mixture of HiLy647-labeled and unlabeled tubulins (Cytoskeleton) in BRB80 buffer (80 mM Pipes/KOH, pH 6.8, 1 mM MgCl_2_, and 1 mM EGTA) supplemented with 1 mM GTP at 37°C for 15 min. After an addition of five volumes of prewarmed BRB80 containing 20 µM taxol and an additional 15 min incubation at 37°C, polymerized MTs were stored at room temperature in the dark for further use. Polymerized MTs were diluted in BRB80 Buffer containing 10 µM taxol and then infused into flow-cells and incubated for 5 min at room temperature for nonspecific adsorption to the coverslip. Subsequently, Blocking Buffer (1 mg/ml casein in P12 buffer [12 mM Pipes/KOH, pH 6.8, 2 mM MgCl_2_, and 1 mM EGTA] with 10 µM taxol) was infused and incubated for another 5 min. Finally, kinesin motors in the motility mixture (0.5–1 µl of S2 cell lysate, 2 mM ATP, 3 mg/ml casein, 10 µM taxol, and oxygen scavenging [1 mM DTT, 1 mM MgCl_2_, 10 mM glucose, 0.2 mg/ml glucose oxidase, and 0.08 mg/ml catalase] in P12 buffer) was added to the flow cell. The flow cells were sealed with molten paraffin wax and imaged by TIRF microscopy using an inverted microscope Ti-E/B (Nikon) equipped with the perfect focus system (Nikon), a 100× 1.49 NA oil immersion TIRF objective (Nikon), three 20-mW diode lasers (488 nm, 561 nm, and 640 nm) and an electron-multiplying charge-coupled device detector (iXon X3DU897; Andor Technology). Imaging of full-length motors was performed by continuous imaging at 200 ms per frame for 1 min, and imaging of truncated motors was performed by continuous imaging at 100 ms per frame for 30 s. Image acquisition was controlled by Nikon Elements software.

### Analysis of single-molecule motility data

Maximum intensity projections were generated from the videos, and kymographs were produced by drawing along motor-decorated tracks (width = 3 pixels) using Nikon Elements software. Run length was calculated by taking the distance moved along the x axis of the kymograph. Dwell time was calculated by taking the time of the event along the y axis of the kymograph. Velocity was defined as the distance on the x axis of the kymograph divided by the time on the y axis of the kymograph. Full-length motors frequently paused during motility events, and thus only the run length and velocity between pauses were analyzed. Motility events that started before image acquisition or finished after image acquisition were included in the analysis, and thus the motility parameters for motors with long dwell times are an underestimate of the true values. Motility data from at least two independent experiments were pooled and analyzed.

Relative landing rates were calculated for truncated motors by normalizing motor protein levels across lysates to ensure equal motor input. The relative landing rate was defined as the number of events per unit MT length per unit time and are reported as number of events (µm/s) ± SEM. Only events that lasted at least five frames (500 ms) were counted. Over 10 different MTs from at least two independent experiments were analyzed for each construct.

### Statistical analysis

Data were analyzed using Stata software. The Shapiro-Wilk test was used to determine whether data were normally distributed. For normally distributed data, Student’s unpaired *t* tests were used to compare individual samples, and one-way ANOVA with post hoc Tukey (a = 0.01 was used to determine whether a significant difference existed) and *t* tests were used to compare multiple samples. Significance levels are represented as follows: *, P = 0.05–0.01; **, P = 0.01–0.001; ***, P = 0.001–0.0001; and ****, P < 0.0001.

### Online supplemental material

Fig. S1 shows antibody staining of larval fillets to analyze the distribution of synaptic proteins in control and Khc mutant neurons. Fig. S2 shows single-molecule motility results obtained using truncated wild-type and mutant kinesin-1 motors. Fig. S3 shows neuron morphogenesis and Golgi outpost distribution in Khc phosphorylation mutants. Fig. S4 shows additional Golgi outpost and hTfR motility parameters. Table S1 shows all oligonucleotides used in this study.

## Supplementary Material

Supplemental Materials (PDF)

## References

[bib1] Allan V.J., Thompson H.M., and McNiven M.A. 2002 Motoring around the Golgi. Nat. Cell Biol. 4:E236–E242. 10.1038/ncb1002-e236 12360306

[bib2] Arthur A.L., Yang S.Z., Abellaneda A.M., and Wildonger J. 2015 Dendrite arborization requires the dynein cofactor NudE. J. Cell Sci. 128:2191–2201. 10.1242/jcs.170316 25908857 PMC4450295

[bib3] Atherton J., Houdusse A., and Moores C. 2013 MAPping out distribution routes for kinesin couriers. Biol. Cell. 105:465–487. 23796124 10.1111/boc.201300012

[bib4] Atherton J., Farabella I., Yu I.M., Rosenfeld S.S., Houdusse A., Topf M., and Moores C.A. 2014 Conserved mechanisms of microtubule-stimulated ADP release, ATP binding, and force generation in transport kinesins. eLife. 3:e03680 10.7554/eLife.03680 25209998 PMC4358365

[bib5] Barkus R.V., Klyachko O., Horiuchi D., Dickson B.J., and Saxton W.M. 2008 Identification of an axonal kinesin-3 motor for fast anterograde vesicle transport that facilitates retrograde transport of neuropeptides. Mol. Biol. Cell. 19:274–283. 10.1091/mbc.E07-03-0261 17989365 PMC2174192

[bib6] Barlan K., Lu W., and Gelfand V.I. 2013 The microtubule-binding protein ensconsin is an essential cofactor of kinesin-1. Curr. Biol. 23:317–322. 10.1016/j.cub.2013.01.008 23394833 PMC3580027

[bib7] Bentley M., and Banker G. 2016 The cellular mechanisms that maintain neuronal polarity. Nat. Rev. Neurosci. 17:611–622. 10.1038/nrn.2016.100 27511065

[bib8] Brendza K.M., Rose D.J., Gilbert S.P., and Saxton W.M. 1999 Lethal kinesin mutations reveal amino acids important for ATPase activation and structural coupling. J. Biol. Chem. 274:31506–31514. 10.1074/jbc.274.44.31506 10531353 PMC3204605

[bib9] Cabantous S., Terwilliger T.C., and Waldo G.S. 2005 Protein tagging and detection with engineered self-assembling fragments of green fluorescent protein. Nat. Biotechnol. 23:102–107. 10.1038/nbt1044 15580262

[bib10] Cai D., McEwen D.P., Martens J.R., Meyhofer E., and Verhey K.J. 2009 Single molecule imaging reveals differences in microtubule track selection between Kinesin motors. PLoS Biol. 7:e1000216 10.1371/journal.pbio.1000216 19823565 PMC2749942

[bib11] Cheng L., Desai J., Miranda C.J., Duncan J.S., Qiu W., Nugent A.A., Kolpak A.L., Wu C.C., Drokhlyansky E., Delisle M.M., 2014 Human CFEOM1 mutations attenuate KIF21A autoinhibition and cause oculomotor axon stalling. Neuron. 82:334–349. 10.1016/j.neuron.2014.02.038 24656932 PMC4002761

[bib12] Edwards S.L., Yu S.C., Hoover C.M., Phillips B.C., Richmond J.E., and Miller K.G. 2013 An organelle gatekeeper function for Caenorhabditis elegans UNC-16 (JIP3) at the axon initial segment. Genetics. 194:143–161. 10.1534/genetics.112.147348 23633144 PMC3632462

[bib13] Farías G.G., Guardia C.M., Britt D.J., Guo X., and Bonifacino J.S. 2015 Sorting of Dendritic and Axonal Vesicles at the Pre-axonal Exclusion Zone. Cell Reports. 13:1221–1232. 10.1016/j.celrep.2015.09.074 26527003 PMC5410646

[bib14] Friedman D.S., and Vale R.D. 1999 Single-molecule analysis of kinesin motility reveals regulation by the cargo-binding tail domain. Nat. Cell Biol. 1:293–297. 10.1038/13008 10559942

[bib15] Fu M.M., and Holzbaur E.L. 2013 JIP1 regulates the directionality of APP axonal transport by coordinating kinesin and dynein motors. J. Cell Biol. 202:495–508. 10.1083/jcb.201302078 23897889 PMC3734084

[bib16] Fu M.M., and Holzbaur E.L. 2014 Integrated regulation of motor-driven organelle transport by scaffolding proteins. Trends Cell Biol. 24:564–574. 10.1016/j.tcb.2014.05.002 24953741 PMC4177981

[bib17] Fu M.M., Nirschl J.J., and Holzbaur E.L.F. 2014 LC3 binding to the scaffolding protein JIP1 regulates processive dynein-driven transport of autophagosomes. Dev. Cell. 29:577–590. 10.1016/j.devcel.2014.04.015 24914561 PMC4109720

[bib18] Gigant B., Wang W., Dreier B., Jiang Q., Pecqueur L., Plückthun A., Wang C., and Knossow M. 2013 Structure of a kinesin-tubulin complex and implications for kinesin motility. Nat. Struct. Mol. Biol. 20:1001–1007. 10.1038/nsmb.2624 23872990

[bib19] Guardia C.M., Farías G.G., Jia R., Pu J., and Bonifacino J.S. 2016 BORC Functions Upstream of Kinesins 1 and 3 to Coordinate Regional Movement of Lysosomes along Different Microtubule Tracks. Cell Reports. 17:1950–1961. 10.1016/j.celrep.2016.10.062 27851960 PMC5136296

[bib20] Gumy L.F., Katrukha E.A., Grigoriev I., Jaarsma D., Kapitein L.C., Akhmanova A., and Hoogenraad C.C. 2017 MAP2 Defines a Pre-axonal Filtering Zone to Regulate KIF1- versus KIF5-Dependent Cargo Transport in Sensory Neurons. Neuron. 94:347–362. 28426968 10.1016/j.neuron.2017.03.046

[bib21] Gyoeva F.K., Bybikova E.M., and Minin A.A. 2000 An isoform of kinesin light chain specific for the Golgi complex. J. Cell Sci. 113:2047–2054. 10806115 10.1242/jcs.113.11.2047

[bib22] Hackney D.D., and Stock M.F. 2000 Kinesin’s IAK tail domain inhibits initial microtubule-stimulated ADP release. Nat. Cell Biol. 2:257–260. 10.1038/35010525 10806475

[bib23] Hammond J.W., Huang C.F., Kaech S., Jacobson C., Banker G., and Verhey K.J. 2010 Posttranslational modifications of tubulin and the polarized transport of kinesin-1 in neurons. Mol. Biol. Cell. 21:572–583. 10.1091/mbc.E09-01-0044 20032309 PMC2820422

[bib24] Hancock W.O. 2014 Bidirectional cargo transport: moving beyond tug of war. Nat. Rev. Mol. Cell Biol. 15:615–628. 10.1038/nrm3853 25118718 PMC5014371

[bib25] Hendricks A.G., Perlson E., Ross J.L., Schroeder H.W. III, Tokito M., and Holzbaur E.L. 2010 Motor coordination via a tug-of-war mechanism drives bidirectional vesicle transport. Curr. Biol. 20:697–702. 10.1016/j.cub.2010.02.058 20399099 PMC2908734

[bib26] Henthorn K.S., Roux M.S., Herrera C., and Goldstein L.S. 2011 A role for kinesin heavy chain in controlling vesicle transport into dendrites in Drosophila. Mol. Biol. Cell. 22:4038–4046. 10.1091/mbc.E10-07-0572 21880894 PMC3204066

[bib27] Huang C.F., and Banker G. 2012 The translocation selectivity of the kinesins that mediate neuronal organelle transport. Traffic. 13:549–564. 10.1111/j.1600-0854.2011.01325.x 22212743 PMC3967410

[bib28] Huang J., Zhou W., Dong W., Watson A.M., and Hong Y. 2009 From the Cover: Directed, efficient, and versatile modifications of the Drosophila genome by genomic engineering. Proc. Natl. Acad. Sci. USA. 106:8284–8289. 10.1073/pnas.0900641106 19429710 PMC2688891

[bib29] Jacobson C., Schnapp B., and Banker G.A. 2006 A change in the selective translocation of the Kinesin-1 motor domain marks the initial specification of the axon. Neuron. 49:797–804. 10.1016/j.neuron.2006.02.005 16543128

[bib30] Kaan H.Y., Hackney D.D., and Kozielski F. 2011 The structure of the kinesin-1 motor-tail complex reveals the mechanism of autoinhibition. Science. 333:883–885. 10.1126/science.1204824 21836017 PMC3339660

[bib31] Kamiyama D., Sekine S., Barsi-Rhyne B., Hu J., Chen B., Gilbert L.A., Ishikawa H., Leonetti M.D., Marshall W.F., Weissman J.S., and Huang B. 2016 Versatile protein tagging in cells with split fluorescent protein. Nat. Commun. 7:11046 10.1038/ncomms11046 26988139 PMC4802074

[bib32] Klinman E., Tokito M., and Holzbaur E.L.F. 2017 CDK5-dependent activation of dynein in the axon initial segment regulates polarized cargo transport in neurons. Traffic. 18:808–824. 10.1111/tra.12529 28941293 PMC5683723

[bib33] Konishi Y., and Setou M. 2009 Tubulin tyrosination navigates the kinesin-1 motor domain to axons. Nat. Neurosci. 12:559–567. 10.1038/nn.2314 19377471

[bib34] Kuijpers M., van de Willige D., Freal A., Chazeau A., Franker M.A., Hofenk J., Rodrigues R.J., Kapitein L.C., Akhmanova A., Jaarsma D., and Hoogenraad C.C. 2016 Dynein Regulator NDEL1 Controls Polarized Cargo Transport at the Axon Initial Segment. Neuron. 89:461–471. 10.1016/j.neuron.2016.01.022 26844830

[bib35] Kulkarni A., Khan Y., and Ray K. 2017 Heterotrimeric kinesin-2, together with kinesin-1, steers vesicular acetylcholinesterase movements toward the synapse. FASEB J. 31:965–974. 10.1096/fj.201600759RRR 27920150

[bib36] Lane J.D., and Allan V.J. 1999 Microtubule-based endoplasmic reticulum motility in Xenopus laevis: activation of membrane-associated kinesin during development. Mol. Biol. Cell. 10:1909–1922. 10.1091/mbc.10.6.1909 10359605 PMC25389

[bib37] Lee T.J., Lee J.W., Haynes E.M., Eliceiri K.W., and Halloran M.C. 2017 The Kinesin Adaptor Calsyntenin-1 Organizes Microtubule Polarity and Regulates Dynamics during Sensory Axon Arbor Development. Front. Cell. Neurosci. 11:107 10.3389/fncel.2017.00107 28473757 PMC5397401

[bib38] Lim A., Rechtsteiner A., and Saxton W.M. 2017 Two kinesins drive anterograde neuropeptide transport. Mol. Biol. Cell. 28:3542–3553. 10.1091/mbc.E16-12-0820 28904207 PMC5683764

[bib39] Lin C.H., Li H., Lee Y.N., Cheng Y.J., Wu R.M., and Chien C.T. 2015 Lrrk regulates the dynamic profile of dendritic Golgi outposts through the golgin Lava lamp. J. Cell Biol. 210:471–483. 10.1083/jcb.201411033 26216903 PMC4523617

[bib40] Maday S., Twelvetrees A.E., Moughamian A.J., and Holzbaur E.L. 2014 Axonal transport: cargo-specific mechanisms of motility and regulation. Neuron. 84:292–309. 10.1016/j.neuron.2014.10.019 25374356 PMC4269290

[bib41] Marks D.L., Larkin J.M., and McNiven M.A. 1994 Association of kinesin with the Golgi apparatus in rat hepatocytes. J. Cell Sci. 107:2417–2426. 7844161 10.1242/jcs.107.9.2417

[bib42] Morfini G.A., You Y.M., Pollema S.L., Kaminska A., Liu K., Yoshioka K., Björkblom B., Coffey E.T., Bagnato C., Han D., 2009 Pathogenic huntingtin inhibits fast axonal transport by activating JNK3 and phosphorylating kinesin. Nat. Neurosci. 12:864–871. 10.1038/nn.2346 19525941 PMC2739046

[bib43] Moua P., Fullerton D., Serbus L.R., Warrior R., and Saxton W.M. 2011 Kinesin-1 tail autoregulation and microtubule-binding regions function in saltatory transport but not ooplasmic streaming. Development. 138:1087–1092. 10.1242/dev.048645 21307100 PMC3042867

[bib44] Nakata T., and Hirokawa N. 2003 Microtubules provide directional cues for polarized axonal transport through interaction with kinesin motor head. J. Cell Biol. 162:1045–1055. 10.1083/jcb.200302175 12975348 PMC2172855

[bib45] Nakata T., Niwa S., Okada Y., Perez F., and Hirokawa N. 2011 Preferential binding of a kinesin-1 motor to GTP-tubulin-rich microtubules underlies polarized vesicle transport. J. Cell Biol. 194:245–255. 10.1083/jcb.201104034 21768290 PMC3144414

[bib46] Niwa S., Lipton D.M., Morikawa M., Zhao C., Hirokawa N., Lu H., and Shen K. 2016 Autoinhibition of a Neuronal Kinesin UNC-104/KIF1A Regulates the Size and Density of Synapses. Cell Reports. 16:2129–2141. 10.1016/j.celrep.2016.07.043 27524618 PMC5432123

[bib47] Ori-McKenney K.M., Jan L.Y., and Jan Y.N. 2012 Golgi outposts shape dendrite morphology by functioning as sites of acentrosomal microtubule nucleation in neurons. Neuron. 76:921–930. 10.1016/j.neuron.2012.10.008 23217741 PMC3523279

[bib48] Pack-Chung E., Kurshan P.T., Dickman D.K., and Schwarz T.L. 2007 A Drosophila kinesin required for synaptic bouton formation and synaptic vesicle transport. Nat. Neurosci. 10:980–989. 10.1038/nn1936 17643120

[bib49] Rolls M.M., and Jegla T.J. 2015 Neuronal polarity: an evolutionary perspective. J. Exp. Biol. 218:572–580. 10.1242/jeb.112359 25696820 PMC4334146

[bib50] Rolls M.M., Satoh D., Clyne P.J., Henner A.L., Uemura T., and Doe C.Q. 2007 Polarity and intracellular compartmentalization of Drosophila neurons. Neural Dev. 2:7 10.1186/1749-8104-2-7 17470283 PMC1868948

[bib51] Ruane P.T., Gumy L.F., Bola B., Anderson B., Wozniak M.J., Hoogenraad C.C., and Allan V.J. 2016 Tumour Suppressor Adenomatous Polyposis Coli (APC) localisation is regulated by both Kinesin-1 and Kinesin-2. Sci. Rep. 6:27456 10.1038/srep27456 27272132 PMC4895226

[bib52] Sanders A.A., and Kaverina I. 2015 Nucleation and Dynamics of Golgi-derived Microtubules. Front. Neurosci. 9:431 10.3389/fnins.2015.00431 26617483 PMC4639703

[bib53] Satoh D., Sato D., Tsuyama T., Saito M., Ohkura H., Rolls M.M., Ishikawa F., and Uemura T. 2008 Spatial control of branching within dendritic arbors by dynein-dependent transport of Rab5-endosomes. Nat. Cell Biol. 10:1164–1171. 10.1038/ncb1776 18758452

[bib54] Schmidt M.R., Maritzen T., Kukhtina V., Higman V.A., Doglio L., Barak N.N., Strauss H., Oschkinat H., Dotti C.G., and Haucke V. 2009 Regulation of endosomal membrane traffic by a Gadkin/AP-1/kinesin KIF5 complex. Proc. Natl. Acad. Sci. USA. 106:15344–15349. 10.1073/pnas.0904268106 19706427 PMC2741253

[bib55] Seno T., Ikeno T., Mennya K., Kurishita M., Sakae N., Sato M., Takada H., and Konishi Y. 2016 Kinesin-1 sorting in axons controls the differential retraction of arbor terminals. J. Cell Sci. 129:3499–3510. 10.1242/jcs.183806 27505885

[bib56] Soppina V., and Verhey K.J. 2014 The family-specific K-loop influences the microtubule on-rate but not the superprocessivity of kinesin-3 motors. Mol. Biol. Cell. 25:2161–2170. 10.1091/mbc.E14-01-0696 24850887 PMC4091829

[bib57] Stone M.C., Roegiers F., and Rolls M.M. 2008 Microtubules have opposite orientation in axons and dendrites of Drosophila neurons. Mol. Biol. Cell. 19:4122–4129. 10.1091/mbc.E07-10-1079 18667536 PMC2555934

[bib58] Sung H.H., Telley I.A., Papadaki P., Ephrussi A., Surrey T., and Rørth P. 2008 Drosophila ensconsin promotes productive recruitment of Kinesin-1 to microtubules. Dev. Cell. 15:866–876. 10.1016/j.devcel.2008.10.006 19081075

[bib59] Tas R.P., Chazeau A., Cloin B.M.C., Lambers M.L.A., Hoogenraad C.C., and Kapitein L.C. 2017 Differentiation between Oppositely Oriented Microtubules Controls Polarized Neuronal Transport. Neuron. 96:1264–1271. 29198755 10.1016/j.neuron.2017.11.018 PMC5746200

[bib60] Taylor C.A., Yan J., Howell A.S., Dong X., and Shen K. 2015 RAB-10 Regulates Dendritic Branching by Balancing Dendritic Transport. PLoS Genet. 11:e1005695 10.1371/journal.pgen.1005695 26633194 PMC4669152

[bib61] Thoresen T., and Gelles J. 2008 Processive movement by a kinesin heterodimer with an inactivating mutation in one head. Biochemistry. 47:9514–9521. 10.1021/bi800747e 18702529 PMC2586147

[bib62] Tortosa E., Adolfs Y., Fukata M., Pasterkamp R.J., Kapitein L.C., and Hoogenraad C.C. 2017 Dynamic Palmitoylation Targets MAP6 to the Axon to Promote Microtubule Stabilization during Neuronal Polarization. Neuron. 94:809–825. 28521134 10.1016/j.neuron.2017.04.042

[bib63] Twelvetrees A.E., Pernigo S., Sanger A., Guedes-Dias P., Schiavo G., Steiner R.A., Dodding M.P., and Holzbaur E.L. 2016 The Dynamic Localization of Cytoplasmic Dynein in Neurons Is Driven by Kinesin-1. Neuron. 90:1000–1015. 10.1016/j.neuron.2016.04.046 27210554 PMC4893161

[bib64] van der Vaart B., van Riel W.E., Doodhi H., Kevenaar J.T., Katrukha E.A., Gumy L., Bouchet B.P., Grigoriev I., Spangler S.A., Yu K.L., 2013 CFEOM1-associated kinesin KIF21A is a cortical microtubule growth inhibitor. Dev. Cell. 27:145–160. 10.1016/j.devcel.2013.09.010 24120883

[bib65] Verhey K.J., and Hammond J.W. 2009 Traffic control: regulation of kinesin motors. Nat. Rev. Mol. Cell Biol. 10:765–777. 10.1038/nrm2782 19851335

[bib66] Verhey K.J., Lizotte D.L., Abramson T., Barenboim L., Schnapp B.J., and Rapoport T.A. 1998 Light chain-dependent regulation of Kinesin’s interaction with microtubules. J. Cell Biol. 143:1053–1066. 10.1083/jcb.143.4.1053 9817761 PMC2132950

[bib67] Williams L.S., Ganguly S., Loiseau P., Ng B.F., and Palacios I.M. 2014 The auto-inhibitory domain and ATP-independent microtubule-binding region of Kinesin heavy chain are major functional domains for transport in the Drosophila germline. Development. 141:176–186. 10.1242/dev.097592 24257625 PMC3865757

[bib68] Winding M., Kelliher M.T., Lu W., Wildonger J., and Gelfand V.I. 2016 Role of kinesin-1-based microtubule sliding in Drosophila nervous system development. Proc. Natl. Acad. Sci. USA. 113:E4985–E4994. 10.1073/pnas.1522416113 27512046 PMC5003282

[bib69] Woehlke G., Ruby A.K., Hart C.L., Ly B., Hom-Booher N., and Vale R.D. 1997 Microtubule interaction site of the kinesin motor. Cell. 90:207–216. 10.1016/S0092-8674(00)80329-3 9244295

[bib70] Woźniak M.J., and Allan V.J. 2006 Cargo selection by specific kinesin light chain 1 isoforms. EMBO J. 25:5457–5468. 10.1038/sj.emboj.7601427 17093494 PMC1679764

[bib71] Yan J., Chao D.L., Toba S., Koyasako K., Yasunaga T., Hirotsune S., and Shen K. 2013 Kinesin-1 regulates dendrite microtubule polarity in Caenorhabditis elegans. eLife. 2:e00133 10.7554/eLife.00133 23482306 PMC3591006

[bib72] Ye B., Zhang Y., Song W., Younger S.H., Jan L.Y., and Jan Y.N. 2007 Growing dendrites and axons differ in their reliance on the secretory pathway. Cell. 130:717–729. 10.1016/j.cell.2007.06.032 17719548 PMC2020851

[bib73] Zheng Y., Wildonger J., Ye B., Zhang Y., Kita A., Younger S.H., Zimmerman S., Jan L.Y., and Jan Y.N. 2008 Dynein is required for polarized dendritic transport and uniform microtubule orientation in axons. Nat. Cell Biol. 10:1172–1180. 10.1038/ncb1777 18758451 PMC2588425

