# Supplementary material for: Autoinhibition of kinesin-1 is essential to the dendrite-specific localization of Golgi outposts
Source: J Cell Biol. 2018 Jul 2;217(7):2531–47. doi: 10.1083/jcb.201708096 (PMC6028532; doi:10.1083/jcb.201708096)
Supplement: Supplemental Materials (PDF) [file JCB_201708096_sm.pdf]

Supplemental material

Kelliher et al., https://doi.org/10.1083/jcb.201708096

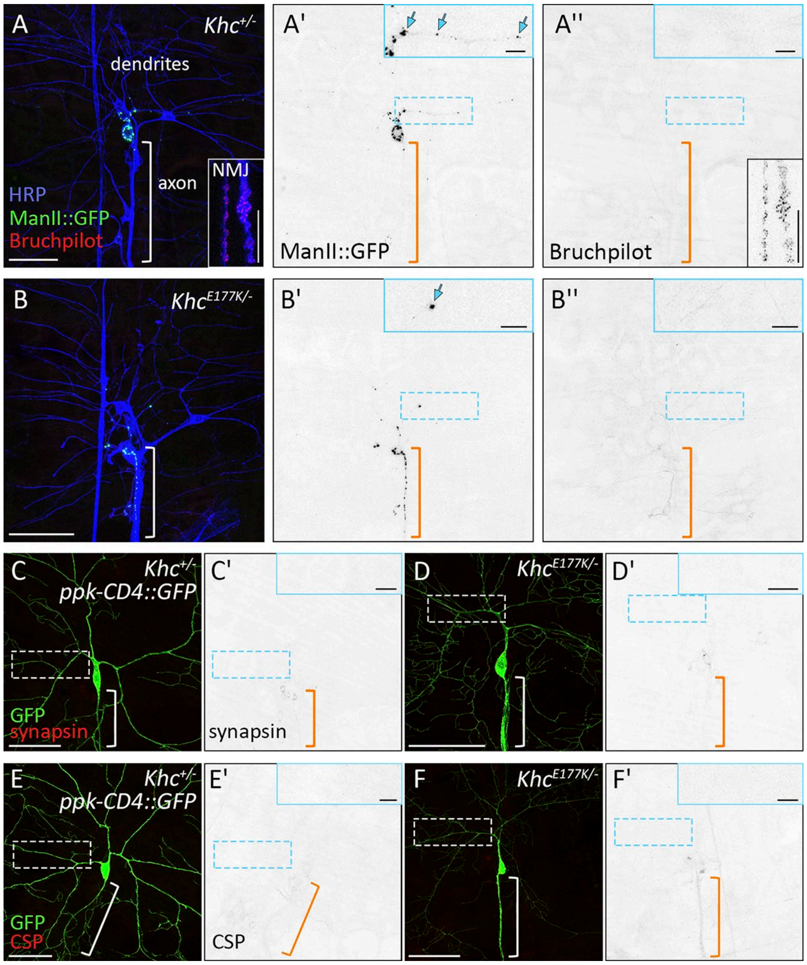

Figure S1. **Localization of Brp, synapsin, and cysteine string protein in *Khc*$^{+/-}$ and *Khc*$^{E177K/-}$ neurons. (A–F)** Brp, synapsin, and cysteine string protein (CSP) are absent from dendrites of *Khc*$^{+/-}$ (A–A'', C, C', E, and E') and *Khc*$^{E177K/-}$ neurons (B–B'', D, D', F, and F'). Insets (A', A'', B', B'', C', D', E', and F') show zoomed view of dendrites (boxed areas), which are devoid of synaptic proteins but contain Golgi outposts (A' and B', arrows). *Khc*$^{E177K/-}$ axons contain mislocalized ManII::GFP-positive Golgi outposts (B and B'). Inset in A and A'' shows Brp at a neuromuscular junction (NMJ), an internal staining control. Neuronal membranes are labeled by anti–horseradish peroxidase (A–B'') and CD4::GFP (C–F'). Bars: (main images) 50 µm; (insets) 20 µm.

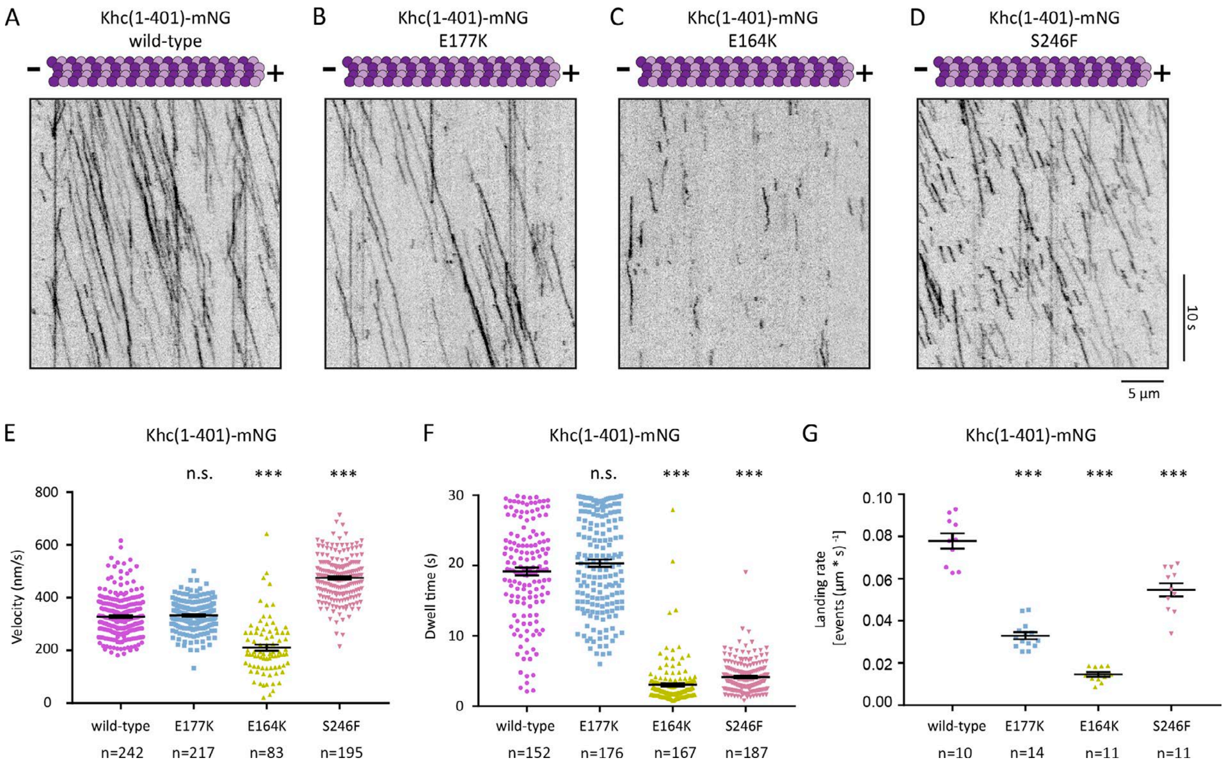

Figure S2. **Effects of mutations on motility of truncated Khc. (A–D)** Representative kymographs showing single-molecule motility of truncated wild-type KHC(1–401) and the indicated mutant versions. All motors were tagged with mNeonGreen at their C termini. Distance is on the x axis, and time is on the y axis. **(E–G)** Quantification of velocities (E), dwell time (F), and landing rate (G) for each population of motors. Note that the truncated Khc(1–401)-S246F::mNeonGreen moves faster than wild-type, but the full-length S246F mutant motor moves more slowly (Fig. 4). Scatterplots display mean ± SEM from at least two independent experiments. ***, P < 0.01 compared with the wild-type motor (calculated by two-tailed *t* test).

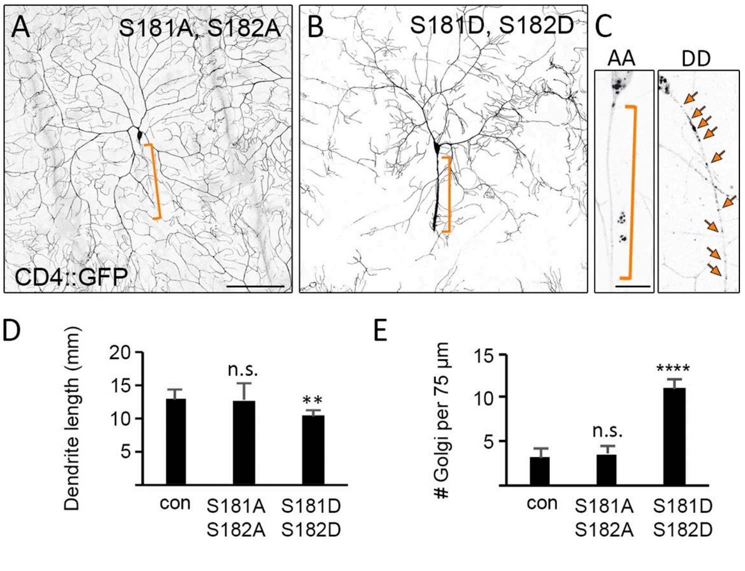

Figure S3. **Effects of Khc phosphomutants on dendrite morphogenesis and Golgi outpost localization. (A, B, and D)** Dendrite arborization is not affected in $Khc^{S181A,S182A/-}$ neurons but is reduced in the phosphomimetic $Khc^{S181D,S182D/-}$ mutants. **(C and E)** Golgi outposts labeled by ManII::GFP (C, arrows) are dendrite-specific in $Khc^{S181A,S182A/-}$ neurons (left), but localize ectopically to axons in $Khc^{S181D,S182D/-}$ neurons (right). Quantification of dendrite length (mean ± SD) of 16 (control), 9 ($Khc^{S181A,S182A/-}$), and 13 ($Khc^{S181D,S182D/-}$) neurons (D) and of Golgi outposts (mean ± SD) in the proximal 75 µm of axons in 9 (control), 8 ($Khc^{S181A,S182A/-}$), and 8 ($Khc^{S181D,S182D/-}$) neurons (E). Brackets indicate axons. **, P < 0.01; ****, P < 0.0001 in comparison with control and evaluated by one-way ANOVA and Tukey post hoc test. Bars: (A and B) 50 µm; (C) 10 µm.

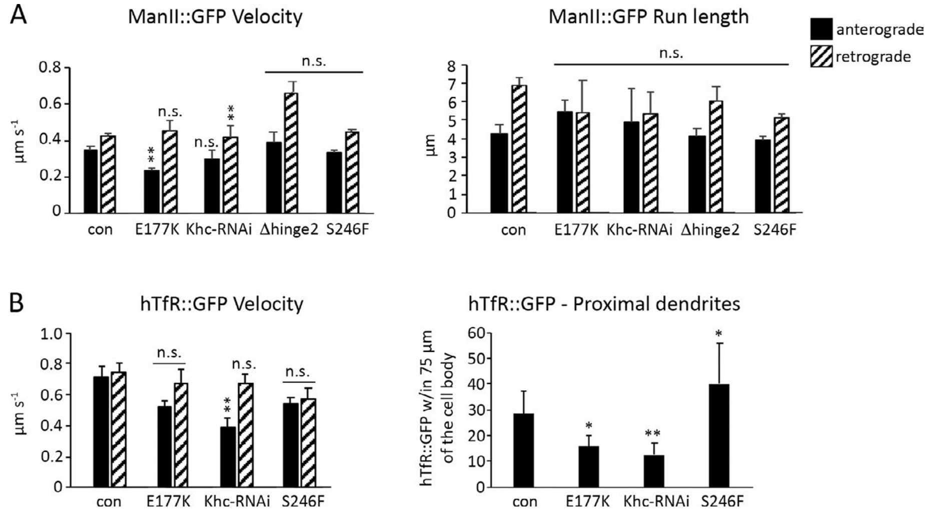

Figure S4. **Golgi outpost and hTfR::GFP motility in control and Khc mutant neurons. (A and B)** Velocity (A and B, mean ± SEM) and run length (A, mean ± SEM) of ManII::GFP (A) and hTfR::GFP (B) in control and mutant genotypes as indicated. The number of hTfR::GFP puncta is reduced in the proximal dendritic arbor. ManII::GFP: control = 338 events in 9 neurons; E177K = 107 events in 10 neurons; Khc-RNAi = 36 events in 11 neurons; Dhinge2 = 22 events in 9 neurons; and S246F = 519 events in 11 neurons. hTfR::GFP: control = 277 events in 11 neurons; E177K = 65 events in 11 neurons; Khc-RNAi = 88 events in 9 neurons; and S246F = 180 events in 9 neurons. *, P = 0.05–0.01; **, P = 0.01–0.001 in comparison with control and evaluated by one-way ANOVA and Tukey post hoc test.

Table S1. **Oligonucleotides and gene fragments used to synthesize constructs used in this study**

Oligonucleotide or gene fragment	Sequence (5′–3′)
Khc73 SQLA substitution PCR primer – F	TTAGCAGTATGTAGTCCAGATGAAGTTATG
Khc73 SQLA substitution PCR primer – R	CTGGCTGCACCCCTTTACATAGGG
Genomic Khc locus sequencing primer – F	TAACCTTTGAGTTCTCTCAGTTGG
Genomic Khc locus sequencing primer – R	ATAGGCGTACTTCAAGTTTTGGTC
Genomic Khc locus sequencing primer – F	CATAAATACTCGAATATGTTGGACG
Genomic Khc locus sequencing primer – R	TACGGTACCAAGGTCTCTTTAGTTTG
Unc104 loop 12 substitution primer – F	CTTTTTCTTGGAAGCCACGTCCGCCAGGGCTGAAAT
Unc104 loop 12 substitution primer – R	AACACCAAGAAGGCAGATCACATCCCCTACCGTGATTC
Unc104 loop 11 substitution primer – F	CTTGGCACCAGTGGAATCCGCTCGCTCGGAACCGGCCAAATC
Unc104 loop 11 substitution primer – R	GGCACTCGCTTGAAGGAGGGAGCCAACATCAACAAGTCGCTGTC
Primer for plasmid + Khc amplification for Gibson Assembly – F	TTCAACAAATCCCTGACTCGGTCCATGTAGATCTCGTAG
Primer for plasmid + Khc amplification for Gibson Assembly – R	TGTCCAAACTAGCCGTTACCTCGCCGGAGGATGTTTTCG
unc104 gene fragment amplification for Gibson forward – F	TCGAAAACATCCTCCGGCGAGGTAACGGCTAGTTTGGAC
unc104 gene fragment amplification for Gibson forward – R	ACTACGAGATCTACATGGACCGAGTCAGGGATTTGTTG
Unc104 gene fragment for loop 8	TCGAAAACATCCTCCGGGGTAACGGCTAGTTTGGACAAATCCTCCACATAGGGACCC AAAAGGGGATGCTCCCTCACACGCAGATTGCCCTTGTTTTTCGGATTCAACAAATCC CTGACTCGGTCCATGTAGATCTCGTAGT
Khc sequencing primer 1 – F	ACTGTTGCCGAGCACAATTC
Khc sequencing primer 2 – F	CTCCAAGAAGGAAATCTTCTGTTTC
Khc sequencing primer 3 – F	AAATCTTCCTCCACCTTGCTG
Khc sequencing primer 4 – F	CTCGATCTCCAGCTTCT
Khc sequencing primer 5 – F	TCGTAGTAGGAGACCTTGATGTG
Khc sequencing primer 6 – F	TCCAAAGTTGCTCTTATTGTTTTTC
Khc sequencing primer 7 – F	ATAGATCTTTCTGGCGGAGATTG
unc104_SKLA substitution PCR primer – F	TTTGGAAGCGCCCTTGACGTACGG
unc104_SKLA substitution PCR primer – R	CTAGCCGTCTCGTCGCCGGAGGATG
K944E mutagenesis PCR primer – F	CGATCTGTGCCTGTGGGCCACG
K944E mutagenesis PCR primer – R	CCGAGCCGATCCGGTCCGGCCAG
E177K mutagenesis PCR primer – F	TTTCGTAGCGCCCTTGACGTACGG
E177K mutagenesis PCR primer – R	CGGTTCGTCTCGTCGCCGGAGGATG
ΔHinge2 deletion PCR primer – F	CTGGTCGTAGTTTACAGC
ΔHinge2 deletion PCR primer – R	TCCAACAAGAAGATCTCC
E177A mutagenesis PCR primer – F	GGCCGTAGCGCCCTTGACGTACGG
E177A mutagenesis PCR primer – R	CGGTTCGTCTCGTCGCCGGAGGATG
S246F mutagenesis PCR primer – F	AAACCTTCTCGGAACCGGCCAAGTC
S246F mutagenesis PCR primer – R	TCAAGACTGGAGCGGAGGGAACCGTT
SS181,182AA mutagenesis PCR primer – F	GGCGACGAACCGTTCCGTAGCGCC
SS181,182AA mutagenesis PCR primer – R	GCCCCGGAGGATGTTTTCGAGGTGATCG
SS181,182DD mutagenesis PCR primer – F	ATCGACGAACCGTTCCGTAGCGCC
SS181,182DD mutagenesis PCR primer – R	GATCCGGAGGATGTTTTCGAGGTGATCG
R947E mutagenesis PCR primer – F	CTTGGCGATCTGTGCCTGTGGGCCACG
R947E mutagenesis PCR primer – R	CCGATCGAGTCCGGCCAGGGTGCAATC
sfGFP amplification PCR primer for Gibson Assembly – F	CTCTCCACGGTGTCCACTC
sfGFP amplification PCR primer for Gibson Assembly – R	TTCGAAGGCGTCCGATTGCC
pIHEU amplification PCR primer for Gibson Assembly – F	ATTAATTAACTAAGCCAGTCGGC

Table S1. **Oligonucleotides and gene fragments used to synthesize constructs used in this study *(Continued)***

**Oligonucleotide or gene fragment**	**Sequence (5′–3′)**
pIHEU amplification PCR primer for Gibson Assembly – R	TGTAATTGAACTGGGAGTGG
sfGFP sequencing primer – F	ATGACGGCACCTACAAGACC
sfGFP sequencing primer – R	TGGTGCAGATGAACTTCAGG
E177R mutagenesis PCR primer – F	GCGCGTAGCGCCCTTGACGTACGG
E177R mutagenesis PCR primer – R	CGGTTCGTCTCGTCGCCGGAGGATG
7×sfGFP11 amplification PCR primer for Gibson Assembly – F	GCGACAGGTGGTGATTGGATCTAGCCGCCGGTGATACC
7×sfGFP11 amplification PCR primer for Gibson Assembly – R	AGGTTAATCCTGTCAACTCGGGTGGCTCTGGAGGTCGT
7×sfGFP11 amplification PCR primer for Gibson Assembly (introduce short linker sequence) – R	GGTGGCTCTGGAGGTCGTGACCACATGGTCCTTCATG
Khc backbone amplification PCR primer for Gibson Assembly – F	ACGACCTCCAGAGCCACCCGAGTTGACAGGATTAACCTGG
Khc backbone amplification PCR primer for Gibson Assembly – R	TAGATCCAATCACCACCTGTCGC
sfGFP gene fragment for Gibson assembly into pIHEU	CTCTCCACAGGTGTCCACTCCCAGTTCAATTACATCTAGAACTAGTGGATCCCCCGG GCTGCAGGAATTCGATATCAAGCTTATCGATACCGTCGACCTCGAGGGGGGGCCCGG TACCGGTGGATCTGGAGGTTCCGGCGGCTCAGGGGGTAGTATGGTGTCCAAGGGCGA GGAGCTGTTCACCGGCGTGGTGCCCATCCTGGTGGAGCTGGATGGCGACGTGAACGG CCACAAGTTCAGCGTGCGCGGCGAGGGCGAGGGCGACGCCACCAACGGCAAGCTGAC CCTGAAGTTCATCTGCACCACCGGCAAGCTGCCCGTGCCCTGGCCCACCCTGGTGAC CACCCTGACCTACGGCGTGCAGTGCTTCAGCCGCTACCCCGATCACATGAAGCAGCA CGATTTCTTCAAGAGCGCCATGCCCGAGGGCTACGTGCAGGAGCGCACCATCAGCTT CAAGGATGACGGCACCTACAAGACCCGCGCCGAGGTGAAGTTCGAGGGCGATACCCT GGTGAACCGCATCGAGCTGAAGGGCATCGATTTCAAGGAGGATGGCAACATCCTGGG CCACAAGCTGGAGTACAACTTCAACAGCCACAACGTGTACATCACCGCCGATAAGCA GAAGAACGGCATCAAGGCCAACTTCAAGATCCGCCACAATGTGGAGGATGGCTCCGT GCAGCTGGCCGATCACTACCAGCAGAACACCCCCATCGGCGACGGCCCAGTGCTGCT GCCCGATAACCACTACCTGAGCACCCAGAGCGTGCTGTCCAAGGACCCCAACGAGAA GCGCGATCACATGGTGCTGCTGGAGTTCGTGACCGCCGCCGGCATCACCCTGGGCAT GGATGAGCTGTACAAGATTAACCCTAGAAAGATAATCATATTGTGAATTAATTAACT AAGCCAGTCGGCAATCGGACGCCTTCGAA
sfGFP(1-10) gene fragment for Gibson assembly into pACUH	GGGAATTCGTTAACAGATCTATGTCCAAAGGAGAAGAACTGTTTACCGGTGTTGTGC CAATTTTGGTTGAACTCGATGGTGATGTCAACGGACATAAGTTCTCAGTGAGAGGCG AAGGAGAAGGTGACGCCACCATTGGAAAATTGACTCTTAAATTCATCTGTACTACTG GTAAACTTCCTGTACCATGGCCGACTCTCGTAACAACGCTTACGTACGGAGTTCAGT GCTTTTCGAGATACCCAGACCATATGAAAAGACATGACTTTTTTAAGTCGGCTATGC CTGAAGGTTACGTGCAAGAAAGAACAATTTCGTTCAAAGATGATGGAAAATATAAAA CTAGAGCAGTTGTTAAATTTGAAGGAGATACTTTGGTTAACCGCATTGAACTGAAAG GAACAGATTTTAAAGAAGATGGTAATATTCTTGGACACAAACTCGAATACAATTTTA ATAGTCATAACGTATACATCACTGCTGATAAGCAAAAGAACGGAATTAAAGCGAATT TCACAGTACGCCATAATGTAGAAGATGGCAGTGTTCAACTTGCCGACCATTACCAAC AAAACACCCCTATTGGAGACGGTCCGGTACTTCTTCCTGATAATCACTACCTCTCAA CACAAACAGTCCTGAGCAAAGATCCAAATGAAAAAGGAACAGGTGGCGGCGGAAGTT AGTCTAGAGGATCTTTGTGAAGG
ManII::GFP amplification primer – F	CACCATGAAGTTAAGTCGCCAG
ManII::GFP amplification primer – R	TTACTTGTACAGCTCGTCCATGC

F, forward; R, reverse.
